# Supplementary figures and images for: Synergistic phase separation of two pathways promotes integrin clustering and nascent adhesion formation
Source: eLife. 2022 Jan 20;11:e72588. doi: 10.7554/eLife.72588 (PMC8791637; doi:10.7554/eLife.72588)

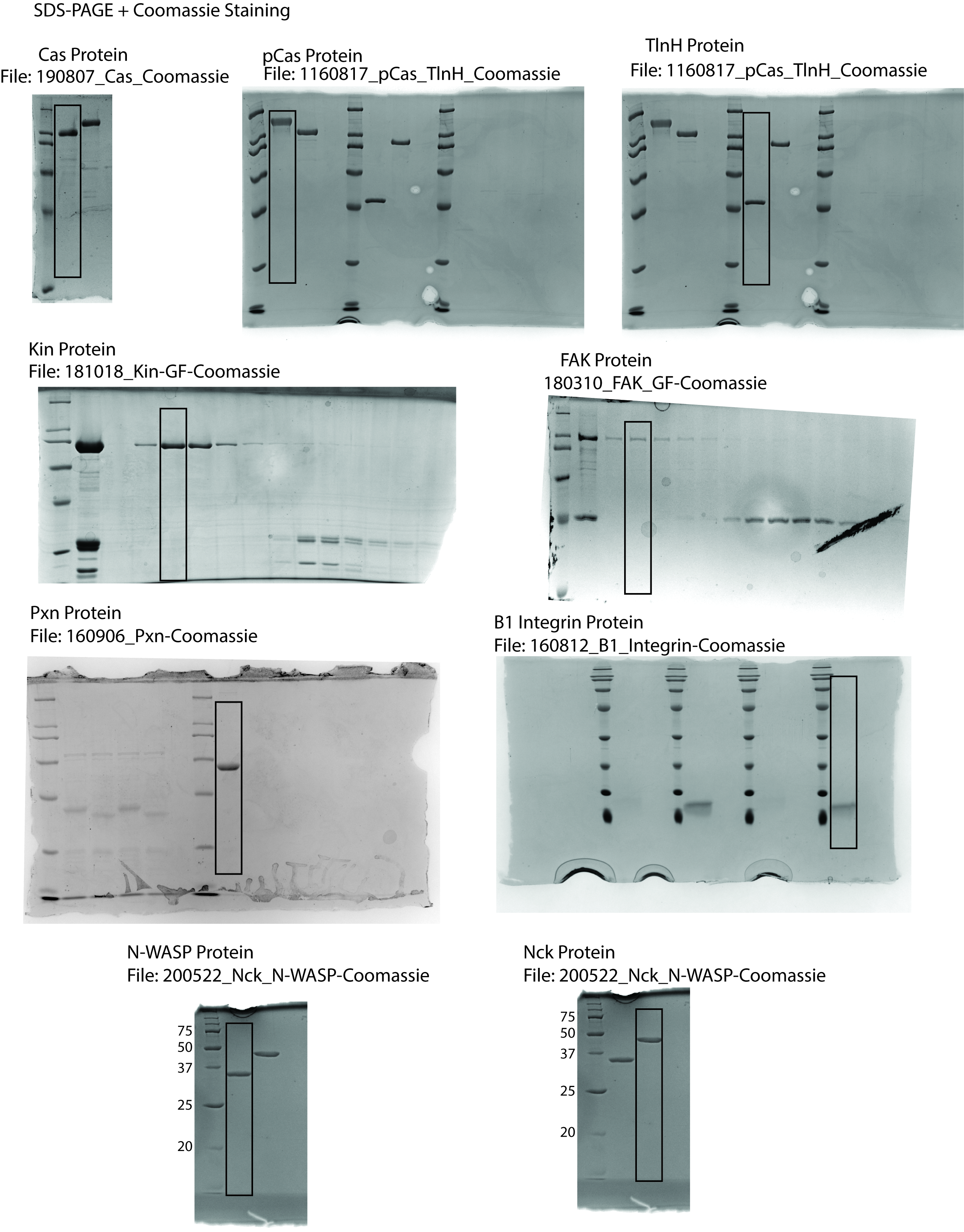

Supplement: Figure 1—figure supplement 2—source data 1. [file elife-72588-fig1-figsupp2-data1.zip › Figure1-figure supplement2-sourcedata/Uncropped_Labeled_Gels_Fig1s2.tif]

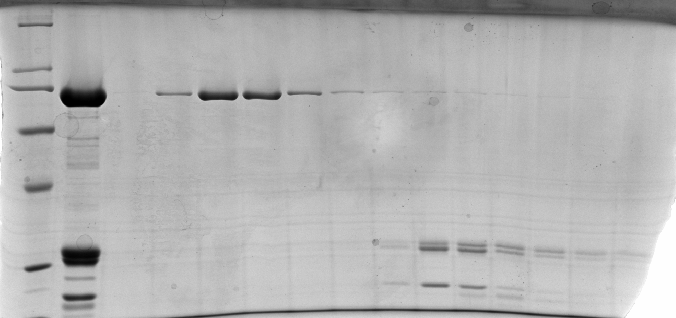

Supplement: Figure 1—figure supplement 2—source data 1. [file elife-72588-fig1-figsupp2-data1.zip › Figure1-figure supplement2-sourcedata/181018_Kin-GF-Coomassie.tiff]

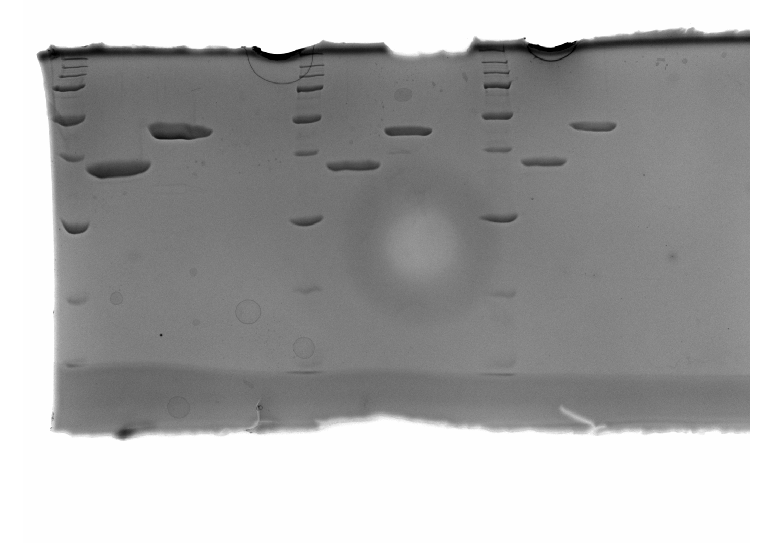

Supplement: Figure 1—figure supplement 2—source data 1. [file elife-72588-fig1-figsupp2-data1.zip › Figure1-figure supplement2-sourcedata/200522_Nck_N-WASP-Coomassie.tif]

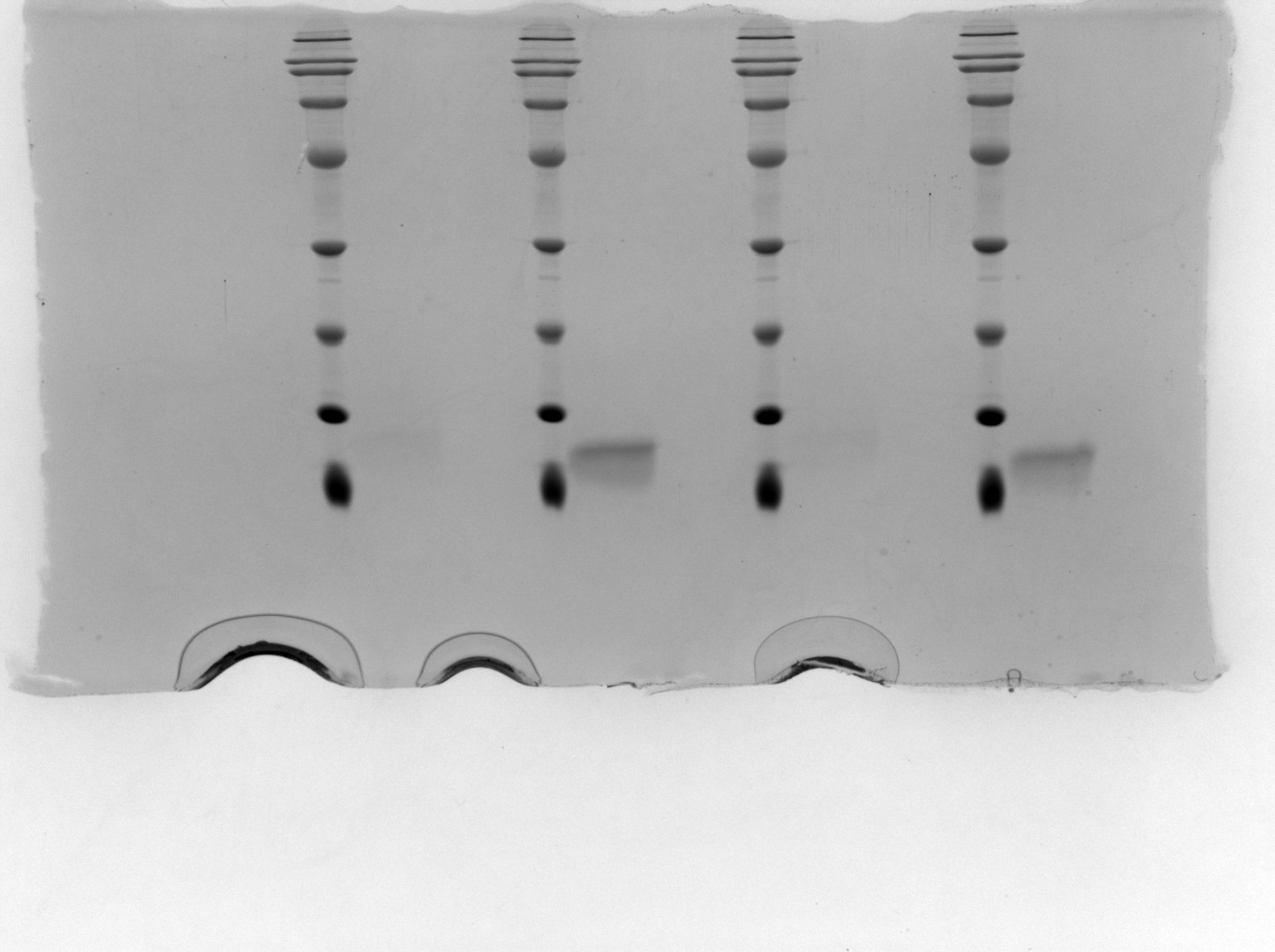

Supplement: Figure 1—figure supplement 2—source data 1. [file elife-72588-fig1-figsupp2-data1.zip › Figure1-figure supplement2-sourcedata/160812_B1_Integrin-Coomassie.tif]

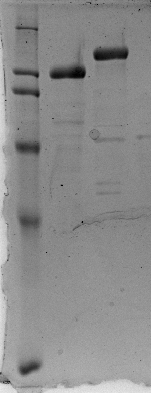

Supplement: Figure 1—figure supplement 2—source data 1. [file elife-72588-fig1-figsupp2-data1.zip › Figure1-figure supplement2-sourcedata/190807_Cas_Coomassie.png]

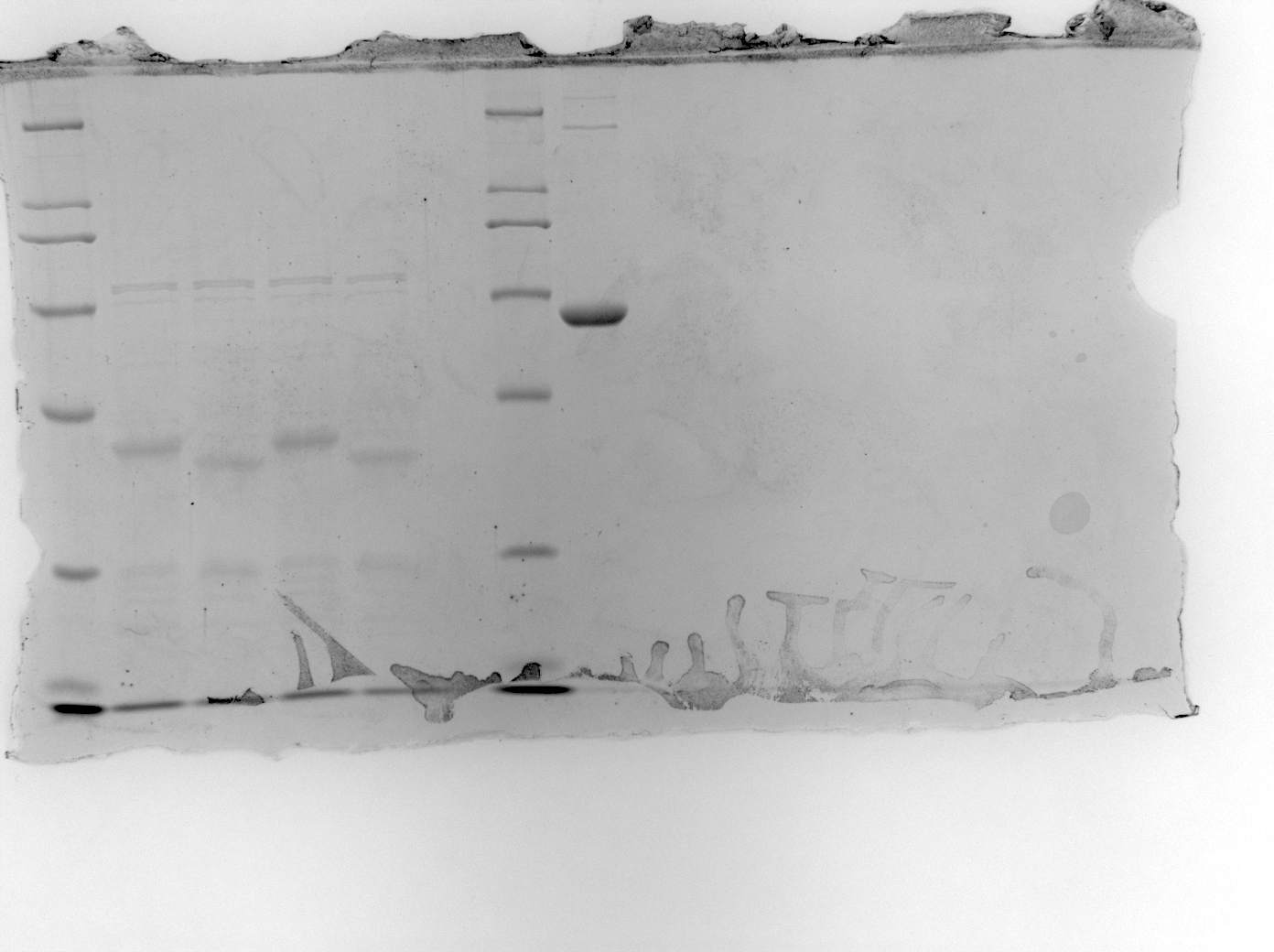

Supplement: Figure 1—figure supplement 2—source data 1. [file elife-72588-fig1-figsupp2-data1.zip › Figure1-figure supplement2-sourcedata/160906_Pxn-Coomassie.tif]

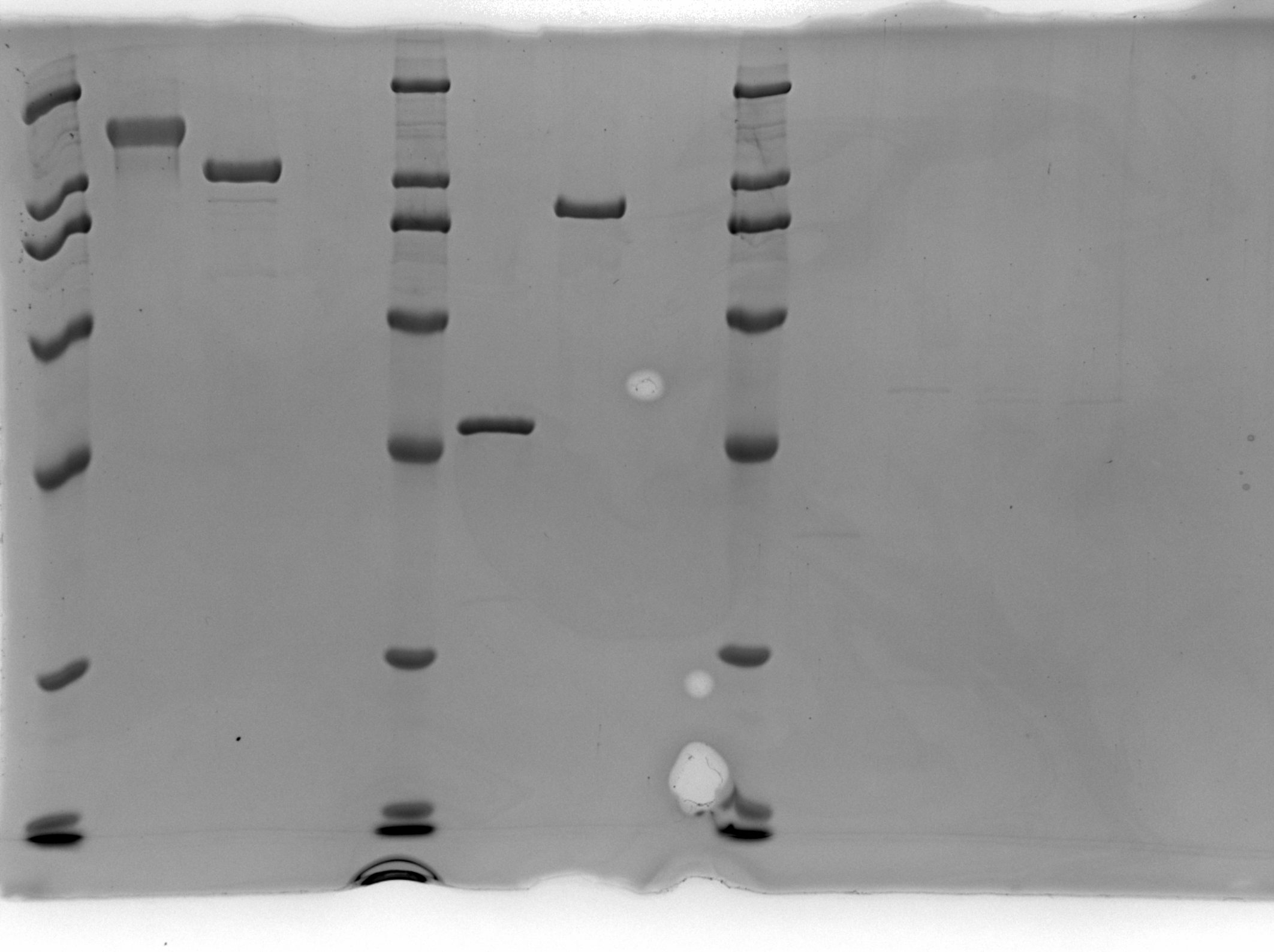

Supplement: Figure 1—figure supplement 2—source data 1. [file elife-72588-fig1-figsupp2-data1.zip › Figure1-figure supplement2-sourcedata/160817_pCas-TlnH-Commassie.tif]

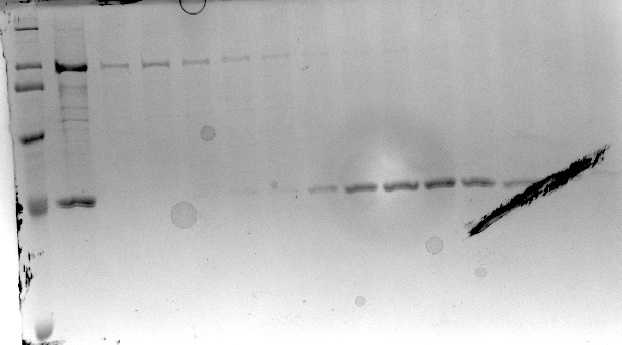

Supplement: Figure 1—figure supplement 2—source data 1. [file elife-72588-fig1-figsupp2-data1.zip › Figure1-figure supplement2-sourcedata/180310_FAK_GF-Coomassie.tiff]

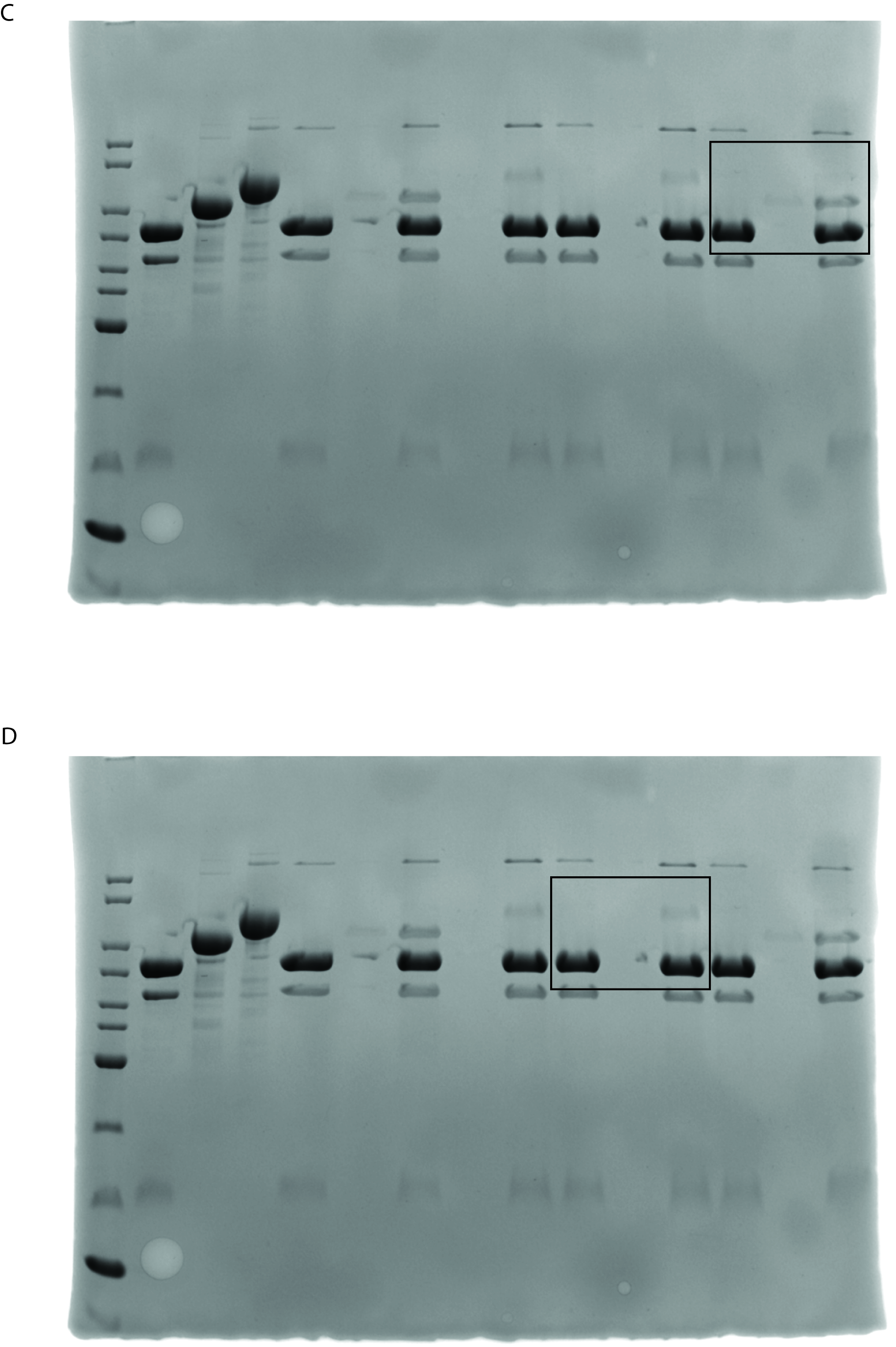

Supplement: Figure 3—figure supplement 2—source data 1. [file elife-72588-fig3-figsupp2-data1.zip › Figure3-figure supplement2 - source data/Uncropped_Labeled_Gels_Fig3s2.tif]

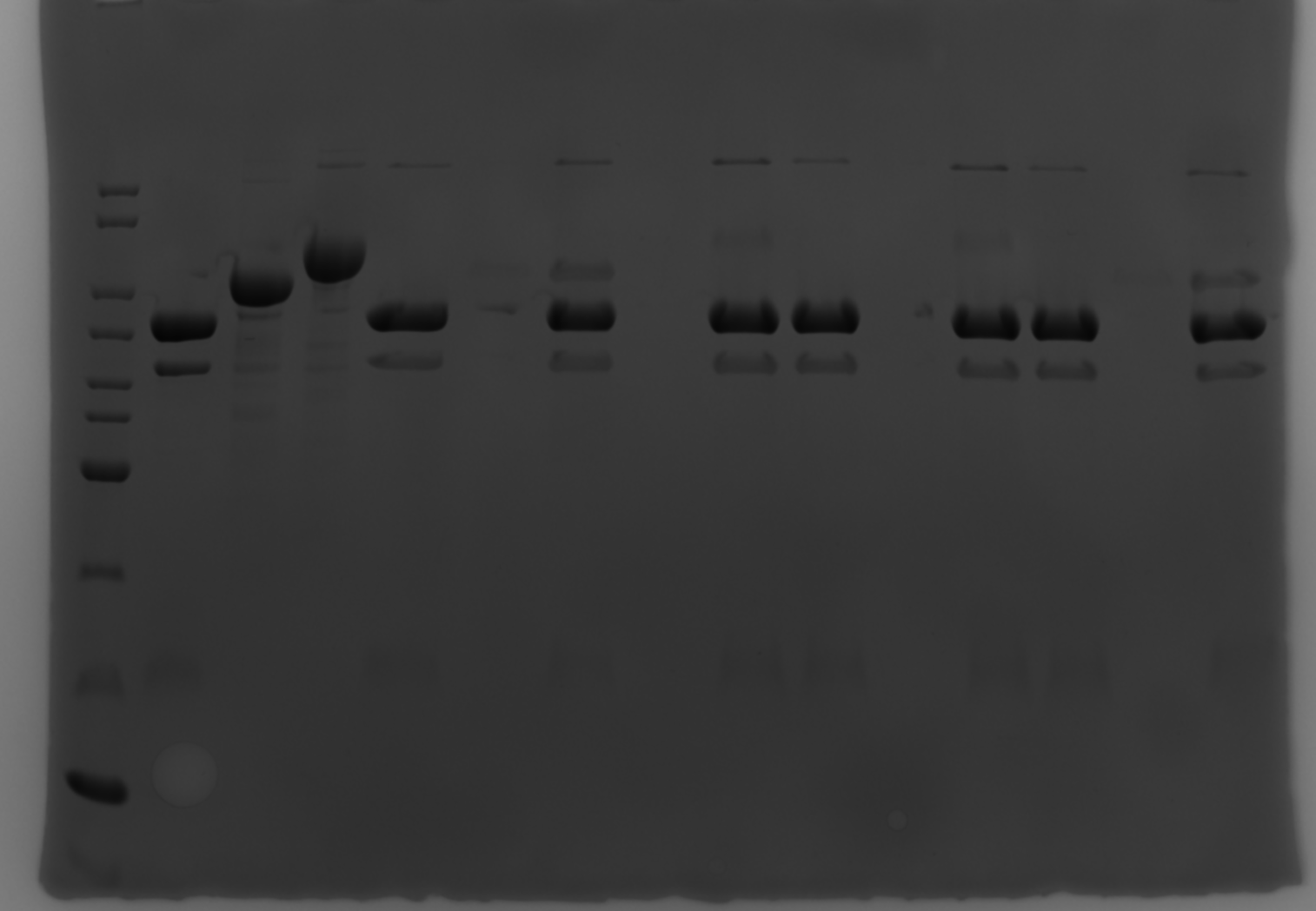

Supplement: Figure 3—figure supplement 2—source data 1. [file elife-72588-fig3-figsupp2-data1.zip › Figure3-figure supplement2 - source data/210511_hisKin-Cas_Pulldown-gel2.tif]

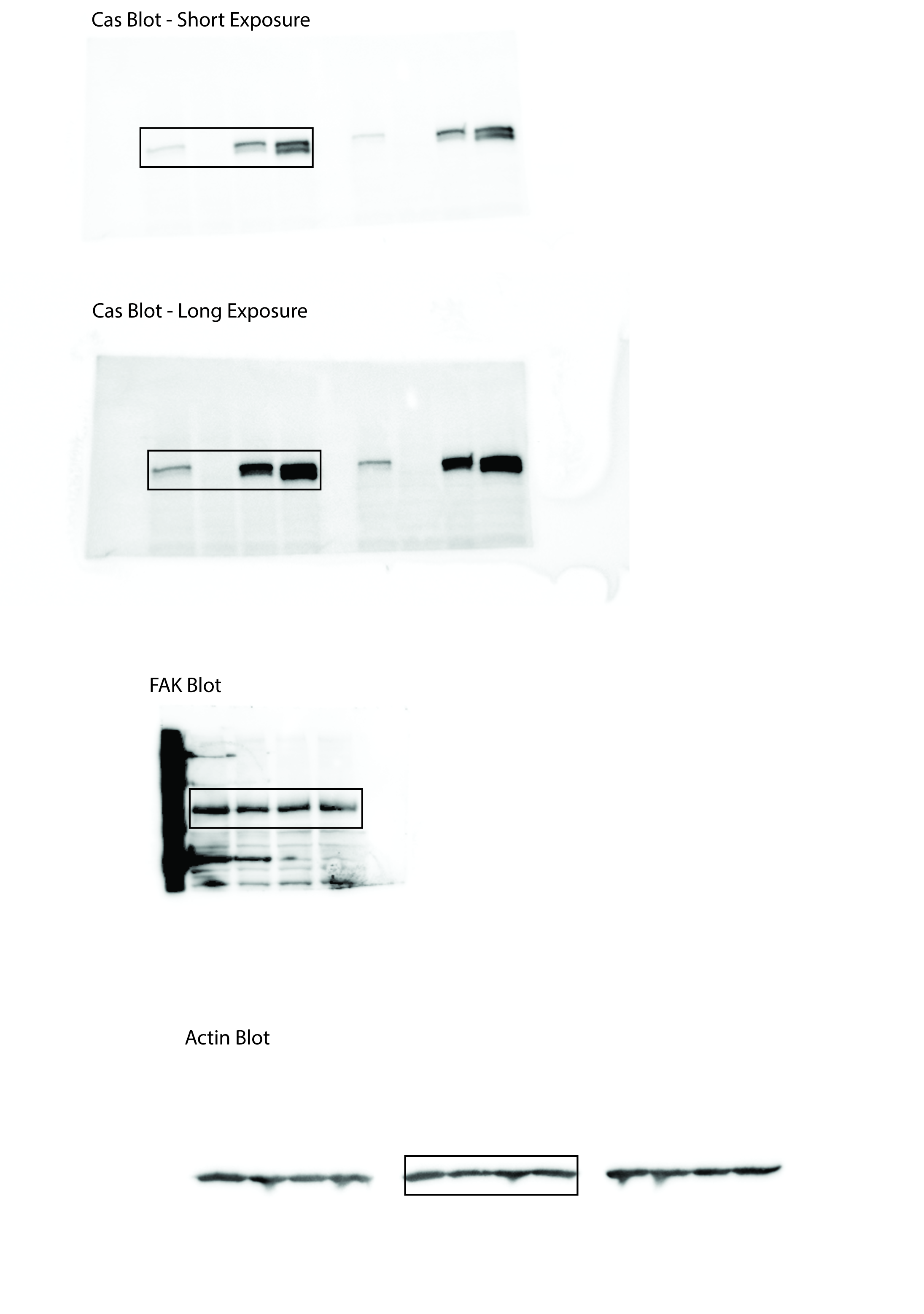

Supplement: Figure 6—figure supplement 3—source data 1. [file elife-72588-fig6-figsupp3-data1.zip › Figure6-figure suppelment3-sourcedata/A/Uncropped_Labeled_Gels_Fig6s2a.tif]

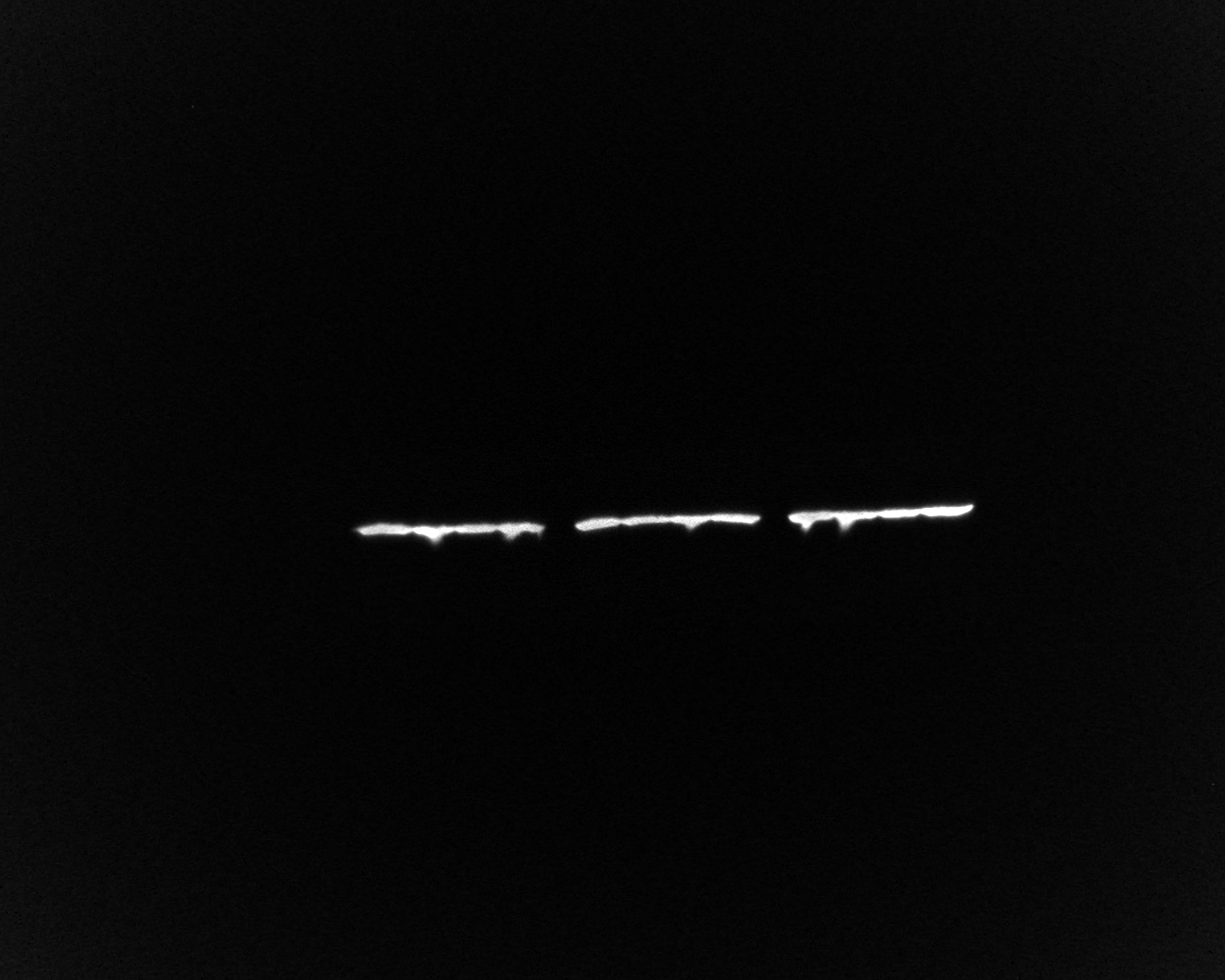

Supplement: Figure 6—figure supplement 3—source data 1. [file elife-72588-fig6-figsupp3-data1.zip › Figure6-figure suppelment3-sourcedata/A/20191217_actinWB 0'5s.tif]

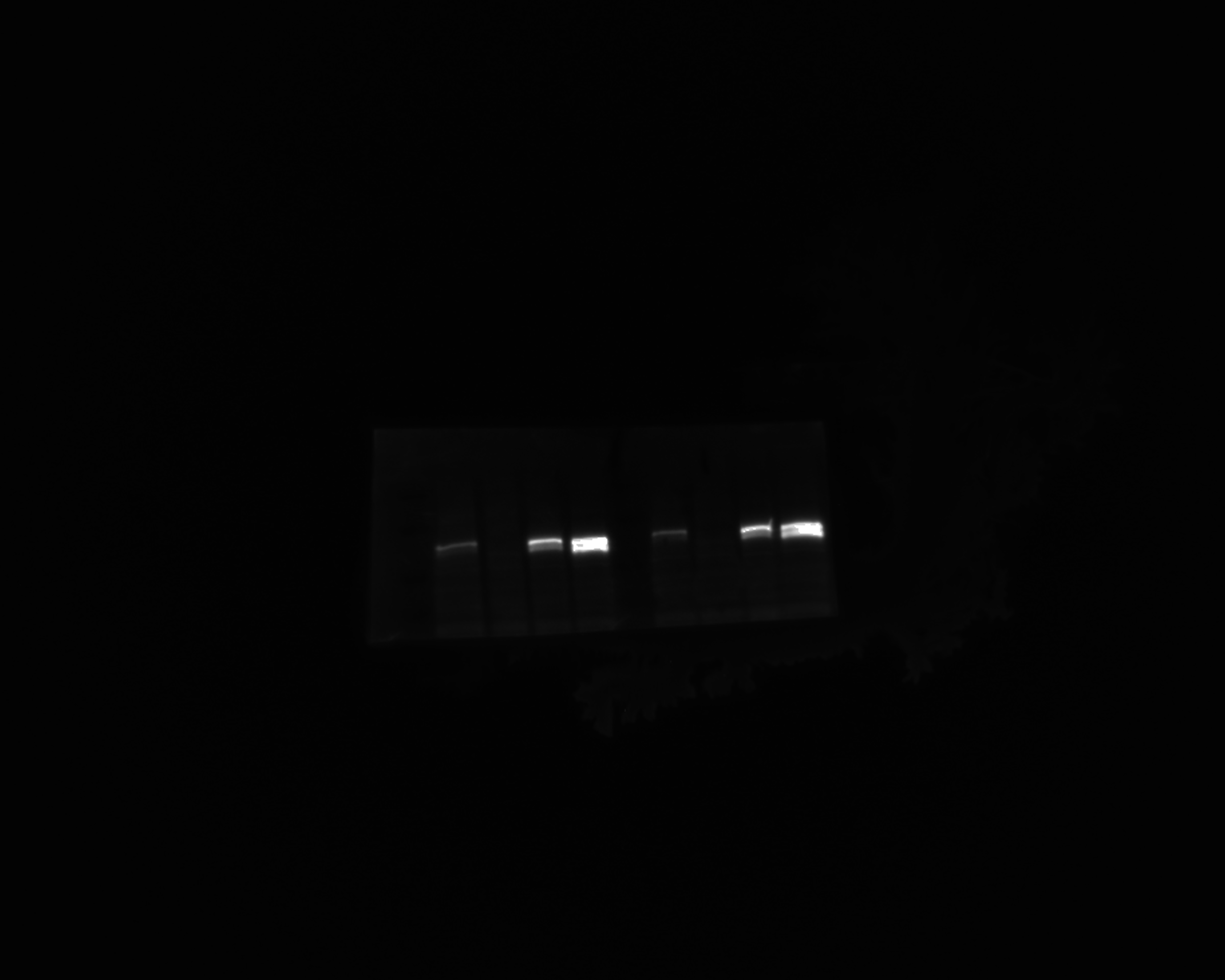

Supplement: Figure 6—figure supplement 3—source data 1. [file elife-72588-fig6-figsupp3-data1.zip › Figure6-figure suppelment3-sourcedata/A/20191217_casWB_60s.tif]

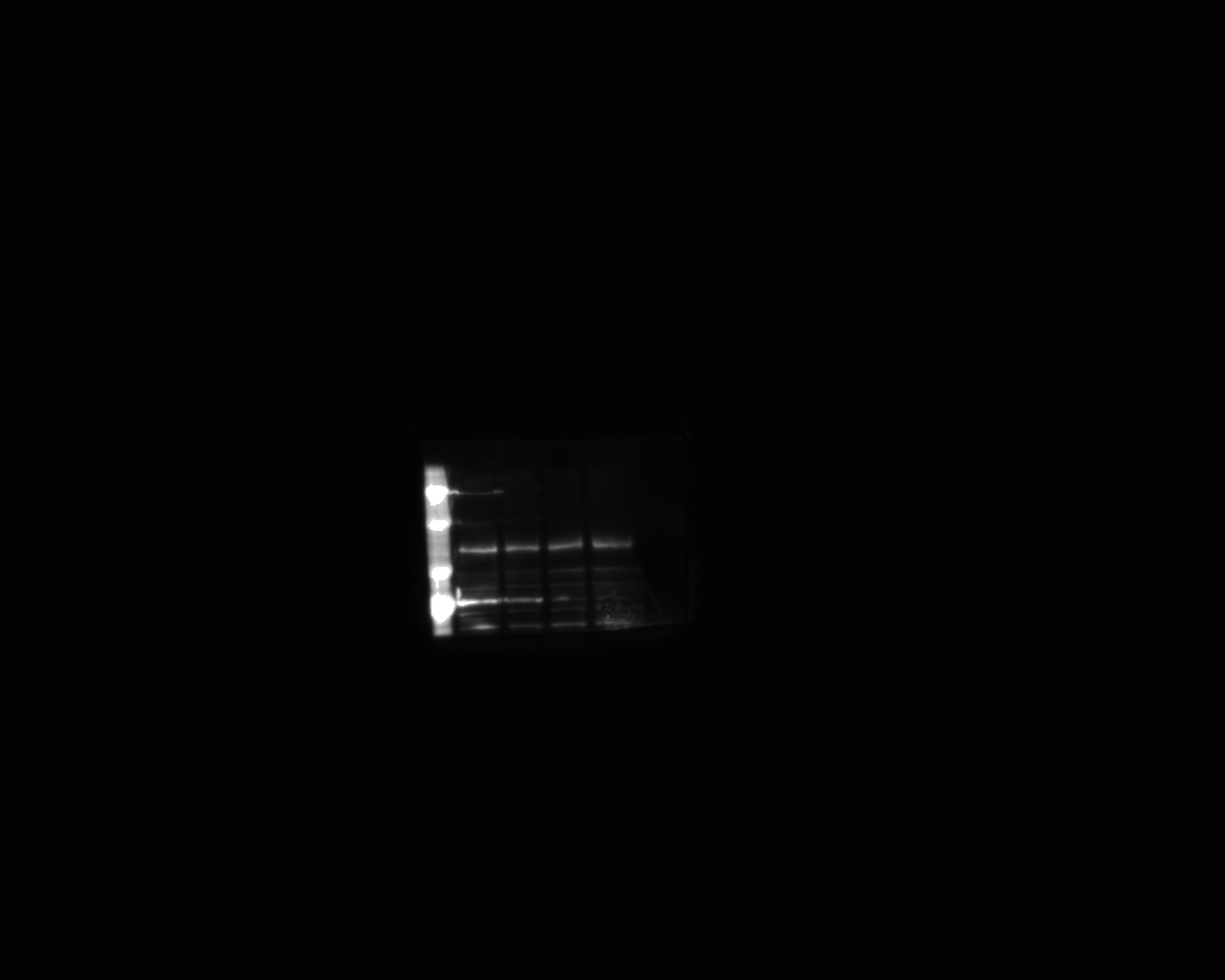

Supplement: Figure 6—figure supplement 3—source data 1. [file elife-72588-fig6-figsupp3-data1.zip › Figure6-figure suppelment3-sourcedata/A/20191217_fakWB_60s.tif]

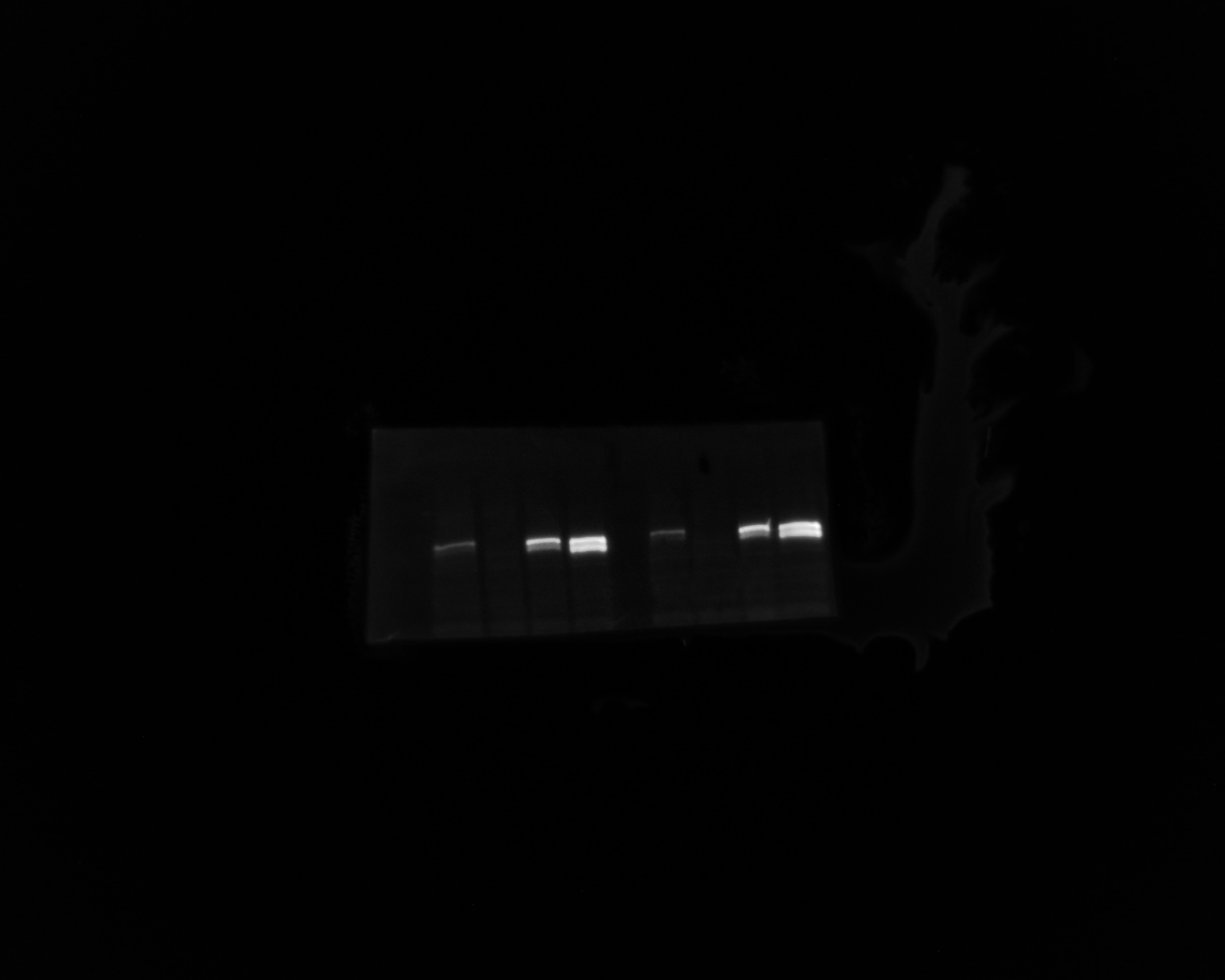

Supplement: Figure 6—figure supplement 3—source data 1. [file elife-72588-fig6-figsupp3-data1.zip › Figure6-figure suppelment3-sourcedata/A/20191217_casWB 30s.tif]

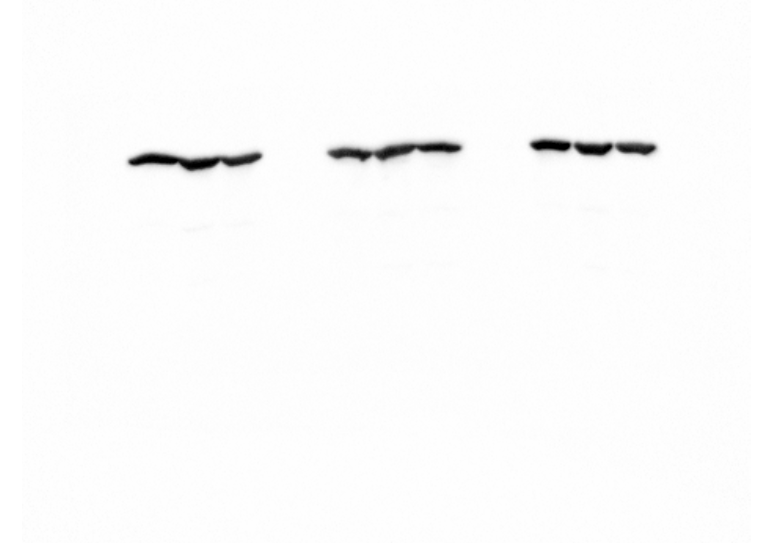

Supplement: Figure 6—figure supplement 3—source data 1. [file elife-72588-fig6-figsupp3-data1.zip › Figure6-figure suppelment3-sourcedata/B/200519_ActinWB_0'5s.tif]

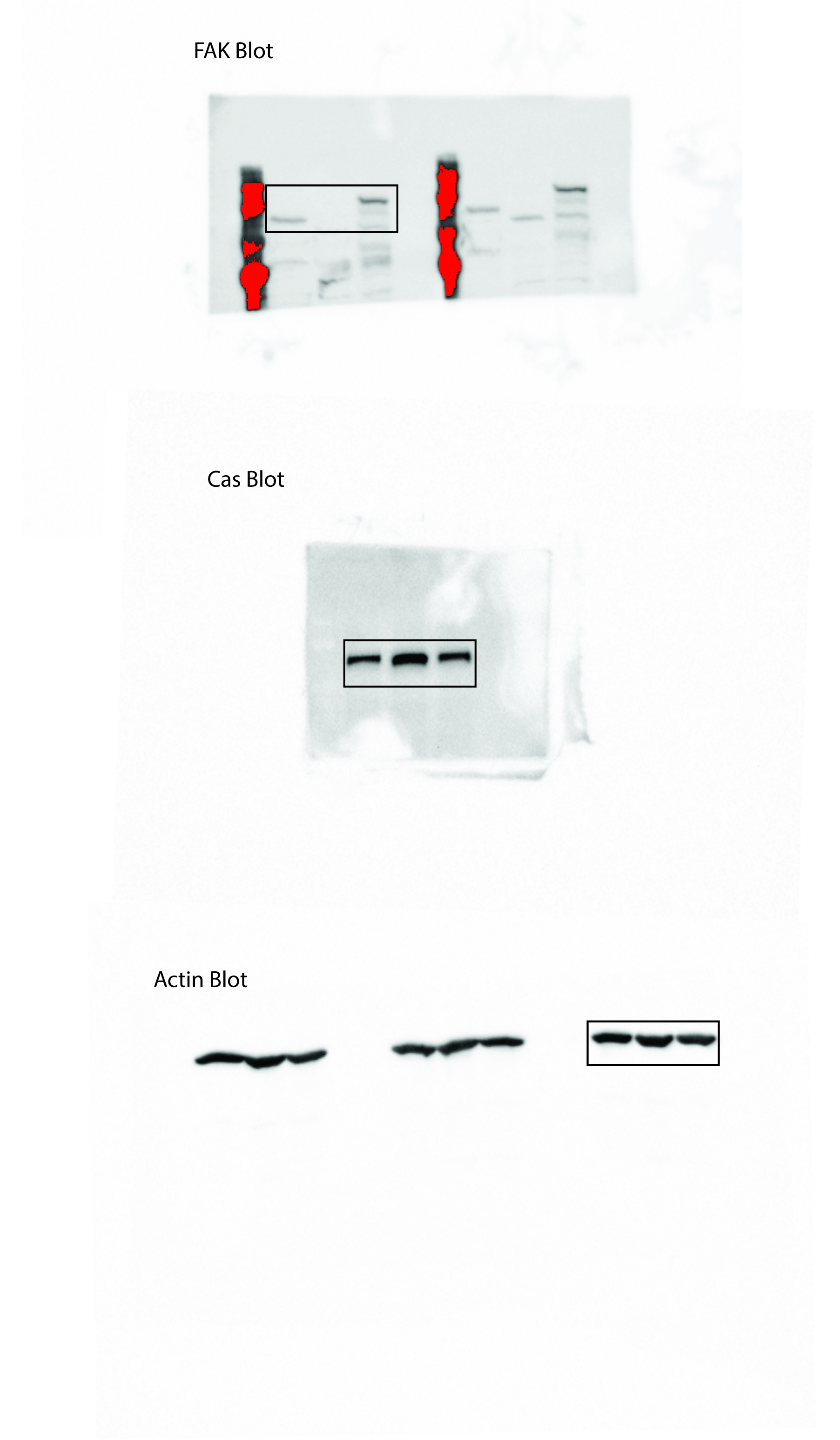

Supplement: Figure 6—figure supplement 3—source data 1. [file elife-72588-fig6-figsupp3-data1.zip › Figure6-figure suppelment3-sourcedata/B/Uncropped_Labeled_Gels_Fig6s2b.tif]

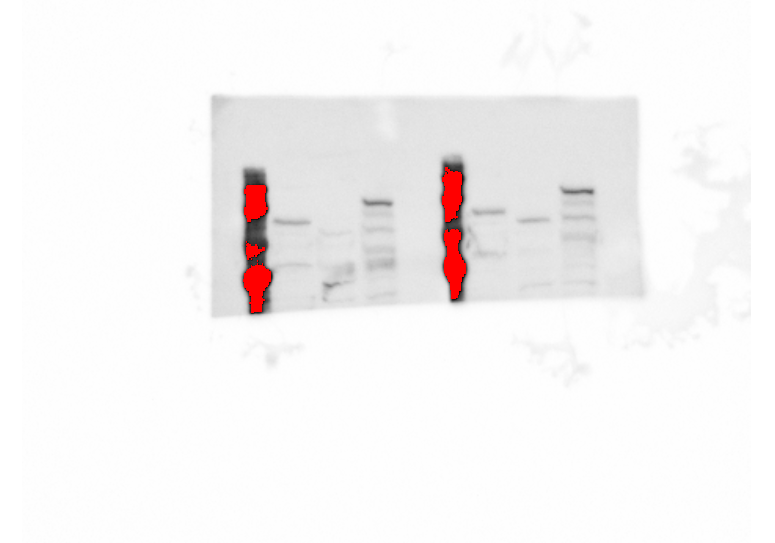

Supplement: Figure 6—figure supplement 3—source data 1. [file elife-72588-fig6-figsupp3-data1.zip › Figure6-figure suppelment3-sourcedata/B/200519_FAKWB_15s.tif]

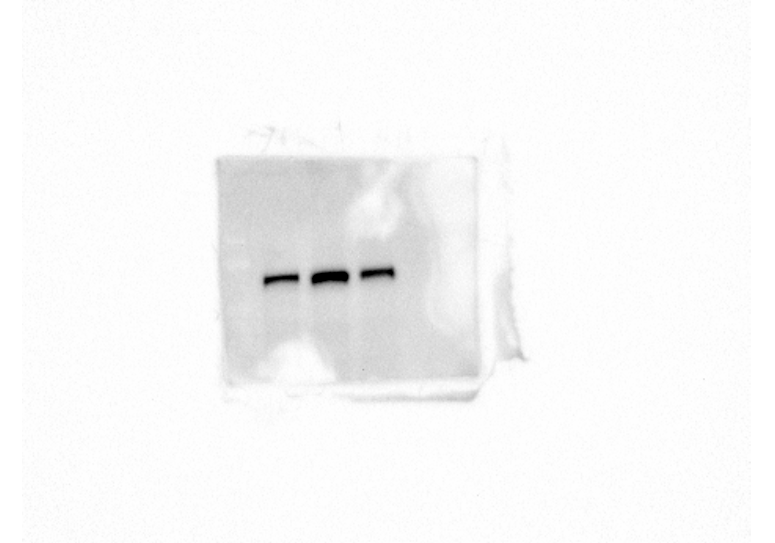

Supplement: Figure 6—figure supplement 3—source data 1. [file elife-72588-fig6-figsupp3-data1.zip › Figure6-figure suppelment3-sourcedata/B/200521_CasWB_10s.tif]

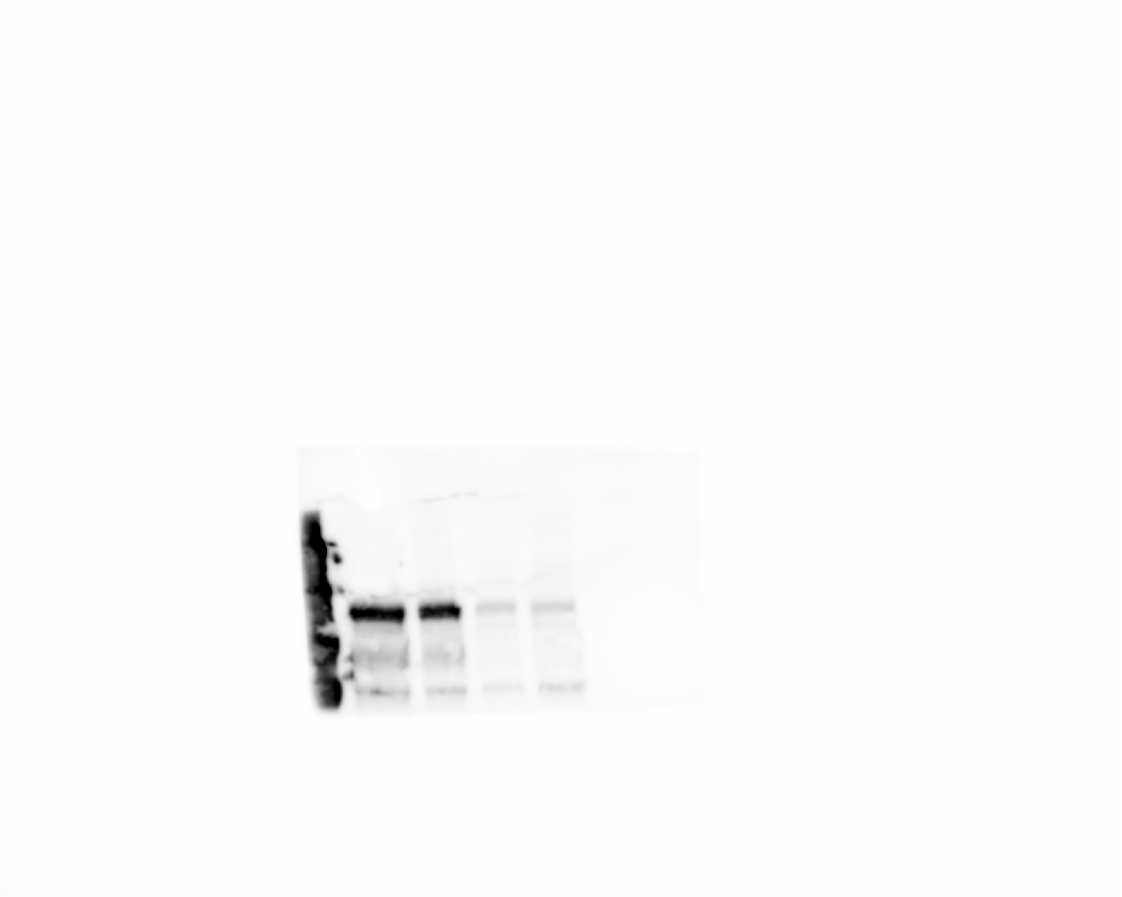

Supplement: Figure 8—source data 1. [file elife-72588-fig8-data1.zip › Figure8-sourcedata/20201201_FAKWB_40s.tif]

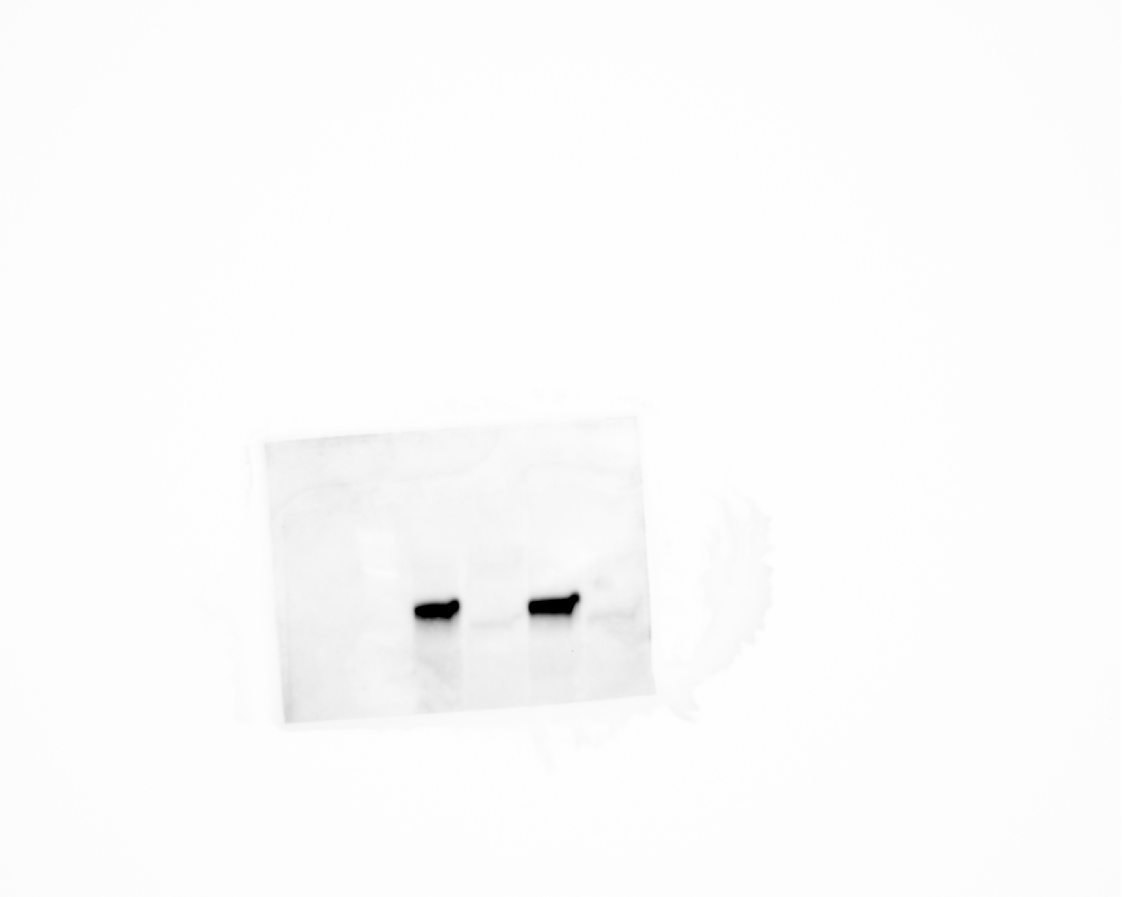

Supplement: Figure 8—source data 1. [file elife-72588-fig8-data1.zip › Figure8-sourcedata/20201201_CasWB_60s.tif]

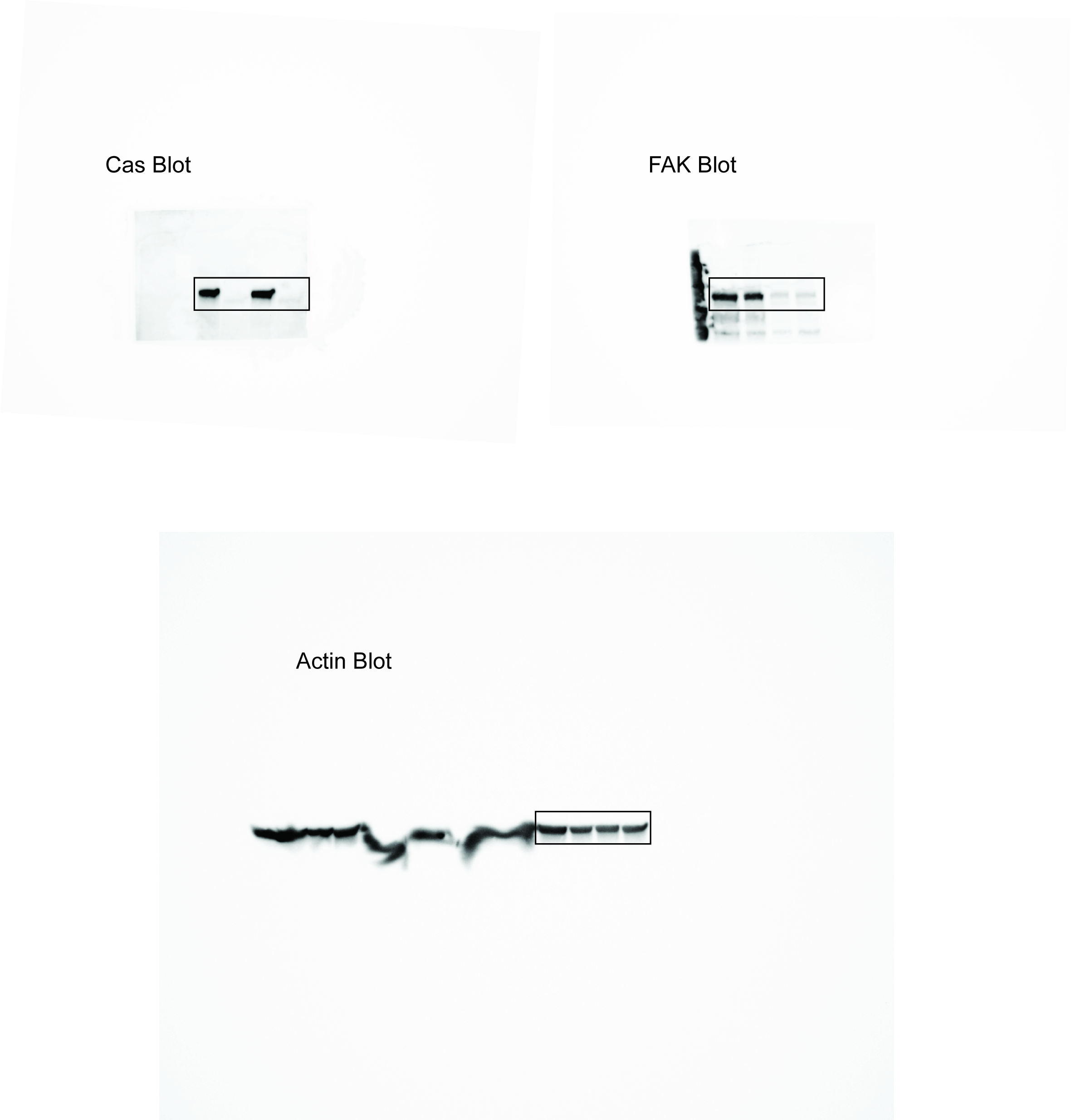

Supplement: Figure 8—source data 1. [file elife-72588-fig8-data1.zip › Figure8-sourcedata/Uncropped_Labeled_Gels_Fig7.tif]

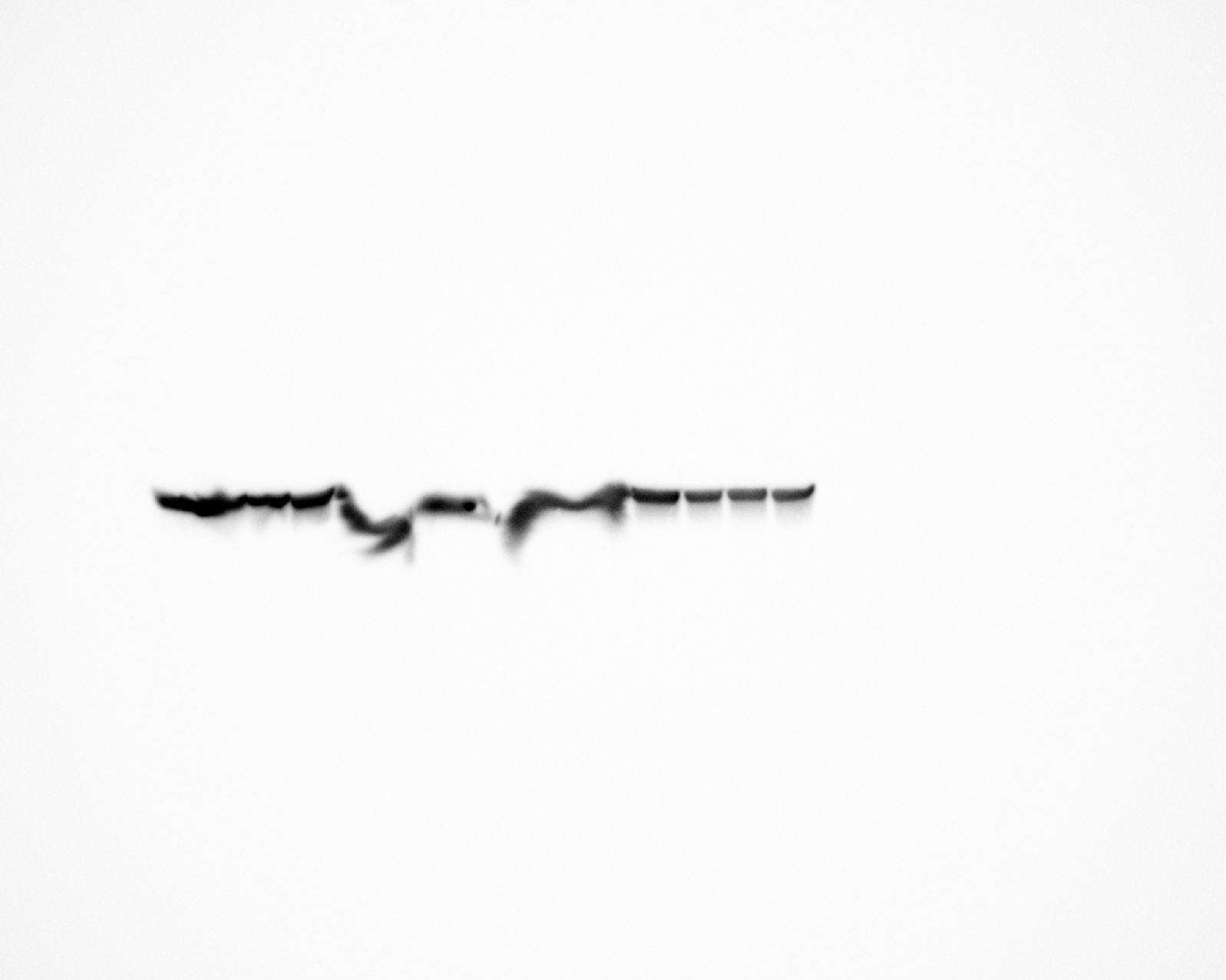

Supplement: Figure 8—source data 1. [file elife-72588-fig8-data1.zip › Figure8-sourcedata/20201201_ActinWB_1s.tif]

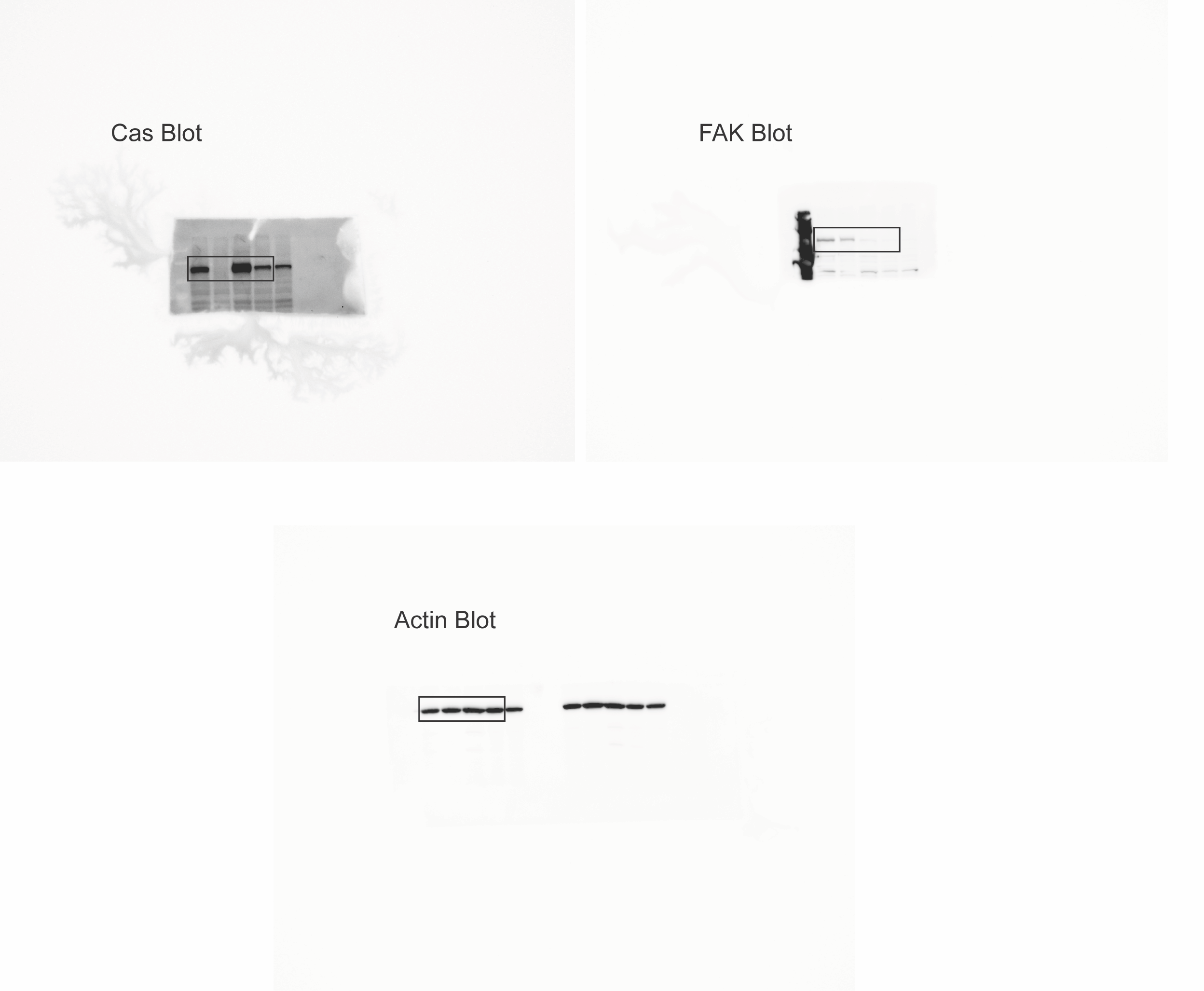

Supplement: Figure 8—figure supplement 1—source data 1. [file elife-72588-fig8-figsupp1-data1.zip › Figure8-figure suppelment1-sourcedata/Uncropped_Labeled_Gels_Fig7s1.tif]

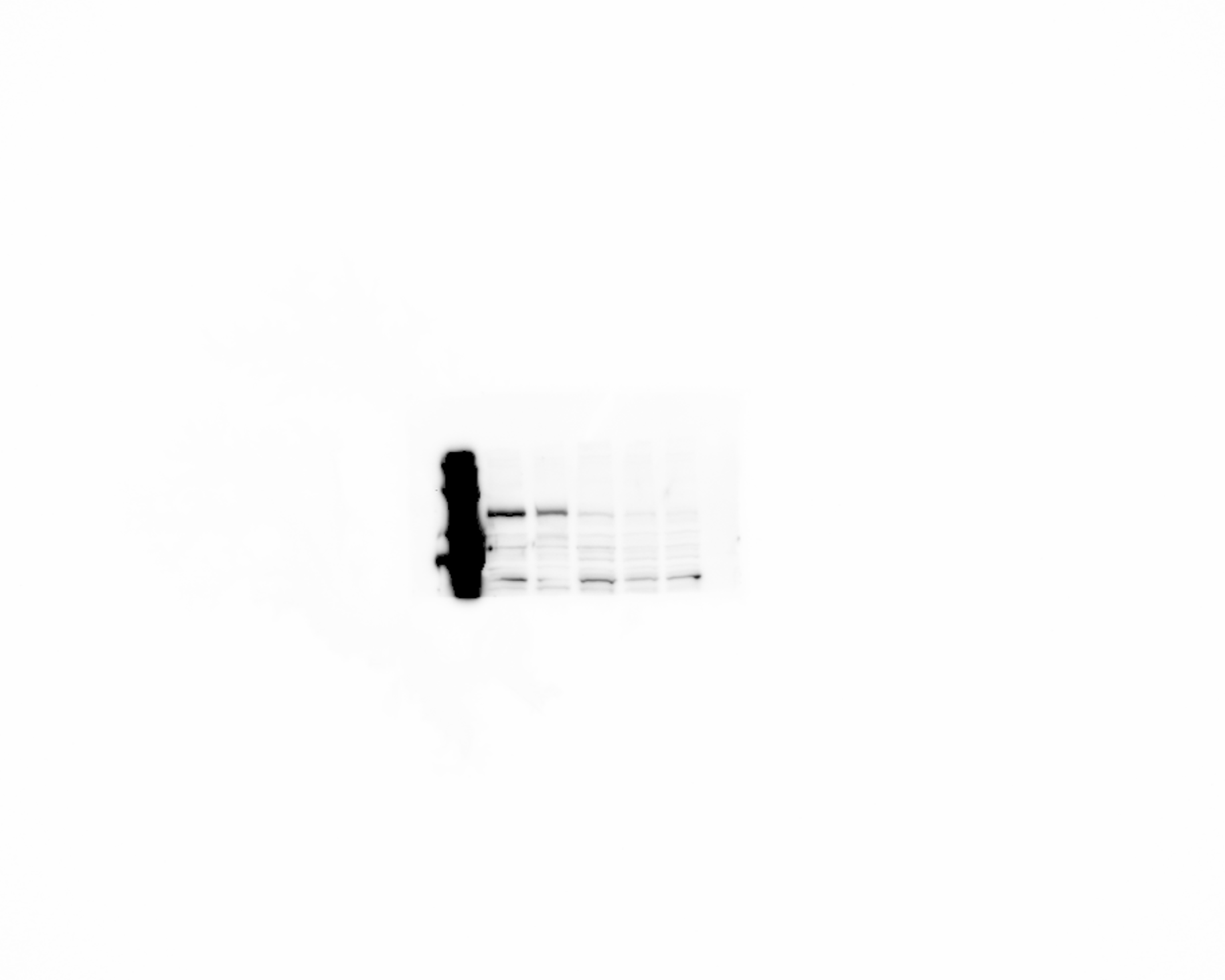

Supplement: Figure 8—figure supplement 1—source data 1. [file elife-72588-fig8-figsupp1-data1.zip › Figure8-figure suppelment1-sourcedata/20191016_fakWB-60s.tif]

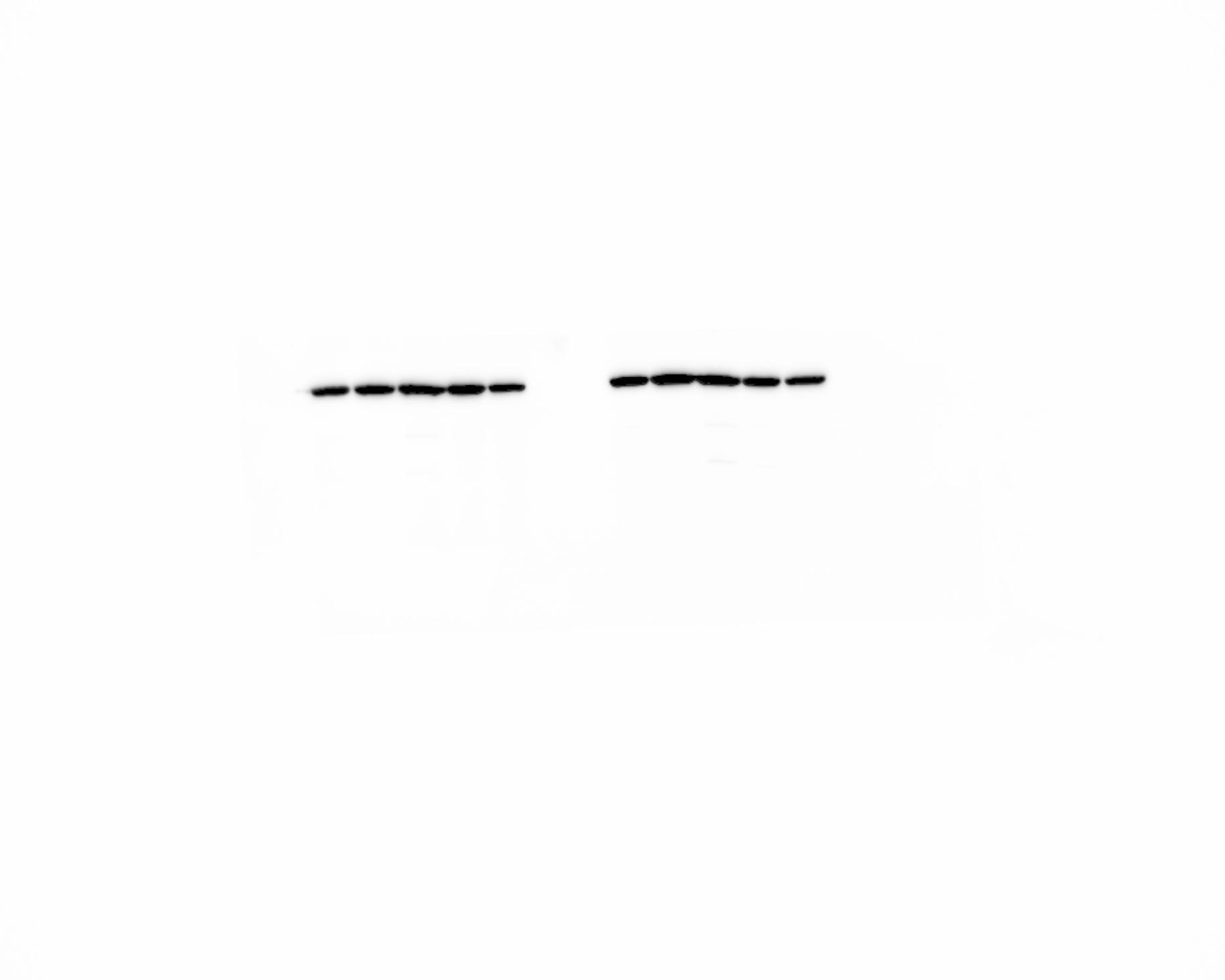

Supplement: Figure 8—figure supplement 1—source data 1. [file elife-72588-fig8-figsupp1-data1.zip › Figure8-figure suppelment1-sourcedata/20191016_actinWB_1s.tif]

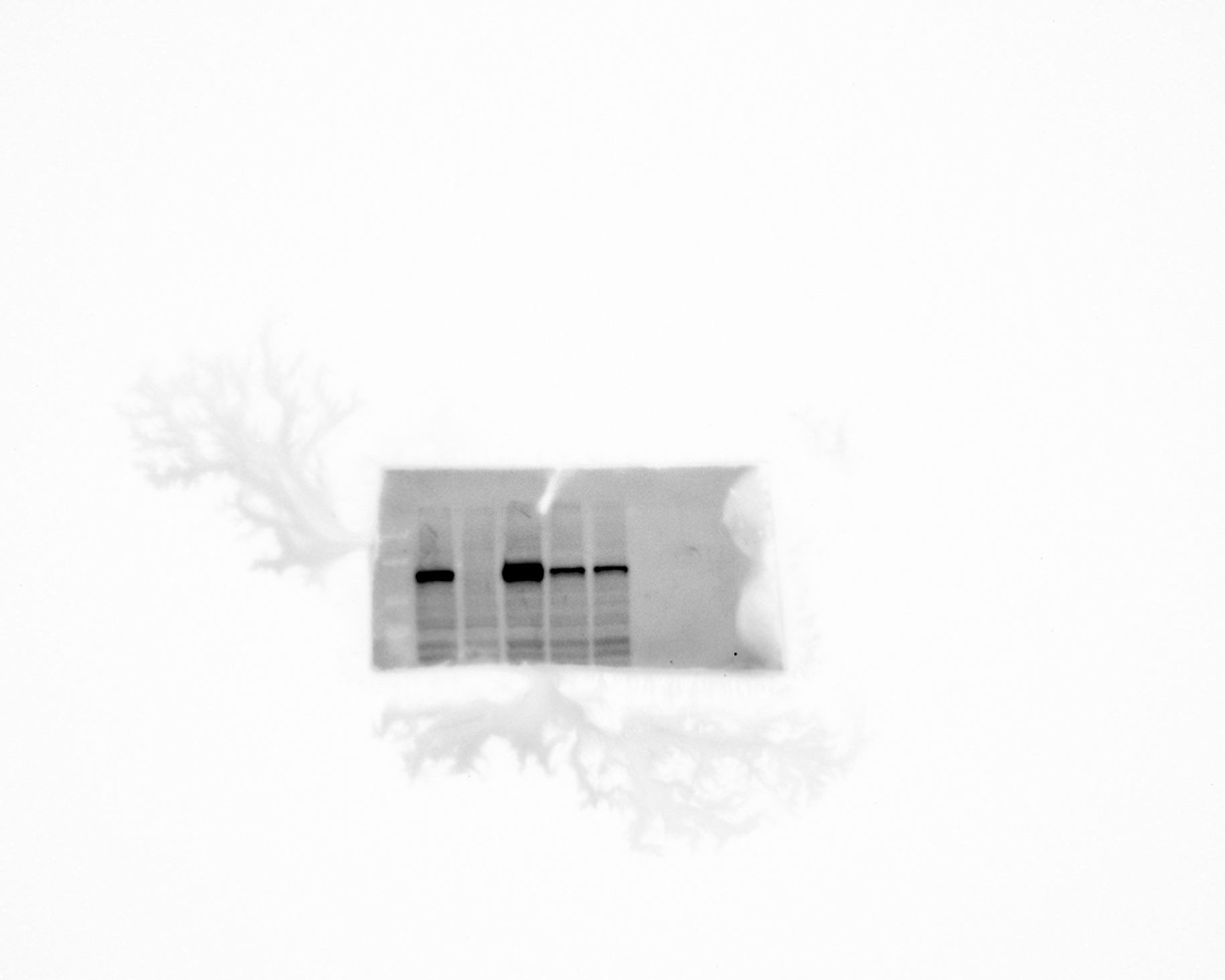

Supplement: Figure 8—figure supplement 1—source data 1. [file elife-72588-fig8-figsupp1-data1.zip › Figure8-figure suppelment1-sourcedata/20191016_casWB_ 60s.tif]
