# Supplementary material for: Synergistic phase separation of two pathways promotes integrin clustering and nascent adhesion formation
Source: eLife. 2022 Jan 20;11:e72588. doi: 10.7554/eLife.72588 (PMC8791637; doi:10.7554/eLife.72588)
Supplement: Supplementary file 1. — Proteins used in this study are in bold.~ indicates the percent recovery was estimated from graph. [file elife-72588-supp1.docx]

**Supplementary File 1**

| **Protein** | **t_1/2_** | **% recovery** | **Citation** | **Notes** |
| --- | --- | --- | --- | --- |
| VASP | 0.7 s | 96% | Lavelin et al., PLoSOne, 2013 | Fig. 7; Hela Cells on FN |
| VASP | 11.1 s | ~75% | Stutchbury et al., JCS, 2017 | Fig 1b + S1a; NIH3T3 on FN |
| **FAK** | 2.1 s | 86% | Lavelin et al., PLoSOne, 2013 | Fig. 7; Hela Cells on FN |
| **FAK** | 9.9 s | ~60% | Stutchbury et al., JCS, 2017 | Fig 1b + S1a; NIH3T3 on FN |
| Zyxin | 0.5 s | 95% | Lavelin et al., PLoSOne, 2013 | Fig. 7; Hela Cells on FN |
| Zyxin | 12 ± 2.5 s | ~75% | Pasapera et al., JCB, 2010 | Fig. 3; MEF on FN |
| Zyxin | 9.4 s | ~80% | Stutchbury et al., JCS, 2017 | Fig 1b + S1a; NIH3T3 on FN |
| **p130Cas** | 14.4 s | ~70% | Stutchbury et al., JCS, 2017 | Fig 1b + S1a; NIH3T3 on FN |
| **p130Cas** | 6.5 ± 0.5 s | ~80% | Branis et al, Scientific Reports, 2017 | Figure 2C; Cas-/- MEFs on FN |
| **p130Cas** | 5.8 ±  0.61 s | ~80% | Donato et al., JBC, 2010 | Figure 4B; Cas-/- MEFs on FN at edge of wounded cell monolayer |
| **Kindlin** | 1.9 s | 63% | Lavelin et al., PLoSOne, 2013 | Fig. 7; Hela Cells on FN |
| **Kindlin** | 23.3 s | ~65% | Stutchbury et al., JCS, 2017 | Fig 1b + S1a; NIH3T3 on FN |
| **Paxillin** | 1.5 s | 70% | Lavelin et al., PLoSOne, 2013 | Fig. 7; Hela Cells on FN |
| **Paxillin** | 15.7 s | ~65% | Stutchbury et al., JCS, 2017 | Fig 1b + S1a; NIH3T3 on FN |
| **Paxillin** | 25 ± 5.9 s | ~90% | Pasapera et al., JCB, 2010 | Fig. 3; MEF on FN |
| $\boldsymbol{\beta}\boldsymbol{1}$**Integrin** | 19.4 ± 2.5 s | 36.5 ± 3.2 % | Stutchbury et al., JCS, 2017 | Fig 1c; NIH3T3 on FN |
| ILK | 29.1 s | ~55% | Stutchbury et al., JCS, 2017 | Fig 1b + S1a; NIH3T3 on FN |
| ILK | 9.2 s | 71% | Lavelin et al., PLoSOne, 2013 | Fig. 7; Hela Cells on FN |
| Talin | 2.2 s | 83% | Lavelin et al., PLoSOne, 2013 | Fig. 7; Hela Cells on FN |
| Talin | 23 ± 3.4 s | ~85% | Pasapera et al., JCB, 2010 | Fig. 3; MEF on FN |
| Talin | 49.4 s | ~50% | Stutchbury et al., JCS, 2017 | Fig 1b + S1a; NIH3T3 on FN |
| $\alpha$-Parvin | 26.0 s | ~55% | Stutchbury et al., JCS, 2017 | Fig 1b + S1a; NIH3T3 on FN |
| $\alpha$-Actinin | 29.6 s | ~75% | Stutchbury et al., JCS, 2017 | Fig 1b + S1a; NIH3T3 on FN |
| $\alpha$-Actinin | 36 ± 0.5 s | ~85% | Pasapera et al., JCB, 2010 | Fig. 3; MEF on FN |
| Vinculin | 1.9 s | 66% | Lavelin et al., PLoSOne, 2013 | Fig. 7; Hela Cells on FN |
| Vinculin | 39.8 s | ~70% | Stutchbury et al., JCS, 2017 | Fig 1b + S1a; NIH3T3 on FN |
| Vinculin | 80 ± 2.9 s | ~70% | Pasapera et al., JCB, 2010 | Fig. 3; MEF on FN |
| Tensin | 59.0 s | ~60% | Stutchbury et al., JCS, 2017 | Fig 1b + S1a; NIH3T3 on FN |
|  |  |  |  |  |
|  |  |  |  |  |
|  |  |  |  |  |
