# Supplementary material for: Synergistic phase separation of two pathways promotes integrin clustering and nascent adhesion formation
Source: eLife. 2022 Jan 20;11:e72588. doi: 10.7554/eLife.72588 (PMC8791637; doi:10.7554/eLife.72588)
Supplement: Supplementary file 2. — Unless the specific domain is indicated in parentheses, studies used full-length protein. Technique abbreviations: ITC: Isothermal Titration Calorimetry; FCS: Fluorescence correlation spectroscopy; Co-IP: Co-immunoprecipitation; SPR: Surface Plasmon Resonance; NMR HSQC: Nuclear Magnetic Resonance Heteronuclear Single Quantum Coherence; AUC: Analytical ultracentrifugation. [file elife-72588-supp2.docx]

**Supplementary File 2.**

| **Protein 1** | **Protein 2** | **K_D_** | **Technique** | **Citation** |
| --- | --- | --- | --- | --- |
| N-WASP | Nck | 54 $\mu M$ | ITC | Banjade et al., PNAS, 2015 |
| Nck | p130Cas | NA | Co-IP | Schlaepfer et al., MCB, 1997 |
| p130Cas (SH3) | FAK (PRR) | 2 $\mu M$ | FCS | Wisniewska et al., JMB, 2005 |
| Paxillin (N-term) | FAK (C-term) | 0.25  ± 0.082 $\mu M$ | SPR/BIAcore | Thomas et al., JBC, 1999 |
| Kindlin (F0) | Paxillin (LIM4) | 200.8 ± 7.2 $\mu M$ | NMR HSQC | Zhu et al., Structure, 2019 |
| Kindlin | Paxillin | 0.205 ± 0.059 μM | ITC | Bottcher et al., JCB, 2017 |
| Kindlin | $\beta1$Integrin (cytosolic) | 20 ± 2 μM | ITC | Li et al., PNAS, 2017 |
| FAK | Nck | NA | Co-IP | Goicoechea et al., IJBCB, 2002 |
| FAK (FERM) | FAK (FERM) | 29 μM | AUC | Brami-Cherrier et al., EMBO, 2014 |
| FAK (FERM) | FAK (FAT) | 0.6 μM | ITC | Brami-Cherrier et al., EMBO, 2014 |
| p130Cas (FAT) | Paxillin (LD1) | 4.2 μM | ITC | Zhang et al., JBC, 2017 |
