# Supplementary material for: Synergistic phase separation of two pathways promotes integrin clustering and nascent adhesion formation
Source: eLife. 2022 Jan 20;11:e72588. doi: 10.7554/eLife.72588 (PMC8791637; doi:10.7554/eLife.72588)
Supplement: Supplementary file 3. [file elife-72588-supp3.docx]

**Supplementary File 3.**

| **Protein** | **Concentration** | **Cell Type** | **Citation** |
| --- | --- | --- | --- |
| WASP | 9000 nM | human peripheral neutrophil | Higgs and Pollard, JCB, 2000 |
| WASP | 2000-8000 nM | T-cell (primed primary CD4+ T cells from 5C.C7 TCR transgenic mice) | Roybal et al., Sci Signaling, 2016 |
| N-WASP | 138 nM | HeLa | Hein et al, Cell, 2015 |
| Nck1 | 180 nM | HeLa | Hein et al, Cell, 2015 |
| p130Cas | 72 nM | HeLa | Hein et al, Cell, 2015 |
| FAK | 40 nM | HeLa | Hein et al, Cell, 2015 |
| FAK | 5-6 nM | NIH3T3 | Brami-Cherrier, EMBO, 2014 |
| Paxillin | 61 nM | HeLa | Hein et al, Cell, 2015 |
| Kindlin | 63 nM | HeLa | Hein et al, Cell, 2015 |
| Integrin $\beta1$ | 188 nM | HeLa | Hein et al, Cell, 2015 |
