# Supplementary material for: Synergistic phase separation of two pathways promotes integrin clustering and nascent adhesion formation
Source: eLife. 2022 Jan 20;11:e72588. doi: 10.7554/eLife.72588 (PMC8791637; doi:10.7554/eLife.72588)
Supplement: Supplementary file 4. — Biexponential and single exponential fits were statistically compared with an extra sum-of-squares F Test to determine the best fit. Values of best fit are shown in table. nd = not determined. For several molecules, the percent recovery could not be accurately fit from these data, as the recovery did not sufficiently plateau within the 90 second experiment. [file elife-72588-supp4.docx]

**Supplementary File 4.**

| **Molecule (bi-exponential)** | **Fast t_1/2_** | **Slow t_1/2_** | **Percent Fast** | **Recovery** |
| --- | --- | --- | --- | --- |
| pCas | 2 s | 39 s | 35% | nd |
| N-WASP | 1 s | 37 s | 30% | 99% |
| FAK (alone) | 6 s | 56 s | 36% | 62% |
| FAK (+Paxillin) | 10 s | 98 s | 21% | nd |
| Paxillin | 7 s | 78 s | 34% | nd |
|  |  |  |  |  |
| **Molecule (single exponential)** | **t_1/2_** | **Recovery** |  |  |
| Nck | 8 s | 39% |  |  |
