# Supplementary material for: Synergistic phase separation of two pathways promotes integrin clustering and nascent adhesion formation
Source: eLife. 2022 Jan 20;11:e72588. doi: 10.7554/eLife.72588 (PMC8791637; doi:10.7554/eLife.72588)
Supplement: Supplementary file 5. — Proteins with multivalent domains shaded grey. Proteins with intrinsically disordered regions longer than 100 amino acids (IDR >100) also indicated. [file elife-72588-supp5.docx]

**Supplementary File 5.**

| **Gene name** | **Protein name** | **UniProtKB Accession** | **IDR >100 aa (Pfam)** | **Domains (Pfam Database)** |
| --- | --- | --- | --- | --- |
| *ACTN4* | actinin, alpha 4 | O43707 | No | 2 CH domains, 4 spectrin repeats, 2 EF hand domains |
| *ILK* | integrin-linked kinase | Q13418 | No | 5 Ankryn repeats |
| *ITGA5* | integrin, alpha 5 (fibronectin receptor, alpha polypeptide) | P08648 | No |  |
| *ITGAV* | integrin, alpha V | P06756 | No |  |
| *ITGB1* | integrin, beta 1 (fibronectin receptor, beta polypeptide, antigen CD29 includes MDF2, MSK12) | P05556 | No |  |
| *LASP1* | LIM and SH3 protein 1 | Q14847 | Yes | 1 Lim domain, 2 nebulin repeats, 1 SH3 domain |
| *PDLIM5* | PDZ and LIM domain 5 | Q96HC4 | Yes | 1 PDZ domain, 3 Lim domain |
| *TGM2* | transglutaminase 2 | P21980 | No | 1 TG N , 2TG C |
| *VASP* | vasodilator-stimulated phosphoprotein | P50552 | Yes | 1 WH1, 1 VASP |
| *VCL* | vinculin | P18206 | Yes | Vinculin head + tail |
| *ACTN1* | actinin, alpha 1 | P12814 | No | 2 CH domains, 4 spectrin repeats, 1 EF hand domains |
| *ARHGEF7* | Rho guanine nucleotide exchange factor (GEF) 7 | Q14155 | Yes | 1 CH, 1 GEF, 1 SH3, 1PH |
| *CNN2* | calponin 2 | Q99439 | No | 1 CH, 3 calponin |
| *DDX18* | DEAD (Asp-Glu-Ala-Asp) box polypeptide 18 | Q9NVP1 | Yes | 1 DEAD, 1 helicase, 1 DUF |
| *FERMT2* | fermitin family member 2 | Q96AC1 | No | 1 N-term, 1 FERM, 1 PH |
| *FHL2* | four and a half LIM domains 2 | Q14192 | No | 4 LIM |
| *FHL3* | four and a half LIM domains 3 | Q13643 | No | 4 LIM |
| *GIT2* | G protein-coupled receptor kinase interacting ArfGAP 2 | Q14161 | Yes | 1 GAP, 2 SHD, 1 CC, 1 C-term |
| *LIMS1* | LIM and senescent cell antigen-like domains 1 | P48059 | No | 5 LIM |
| *LPP* | LIM domain containing preferred translocation partner in lipoma | Q93052 | Yes | 3 LIM |
| *PALLD* | palladin, cytoskeletal associated protein | Q8WX93 | Yes | 5 I-SET |
| *PARVA* | parvin, alpha | Q9NVD7 | No | 2 CH |
| *PDLIM7* | PDZ and LIM domain 7 (enigma) | Q9NR12 | Yes | 1 PDZ, 3 LIM |
| *PLS3* | plastin 3 | P13797 | No | 1 Efy, 4 CH |
| *PTK2* | protein tyrosine kinase 2, focal adhesion kinase | Q05397 | Yes | 1 FERM, 1 Kinase, 1 FAT |
| *PXN* | paxillin | P49023 | Yes | 1 Paxillin, 4 LIM |
| *RSU1* | Ras suppressor protein 1 | Q15404 | No | 4 LRR |
| *TES* | testis derived transcript (3 LIM domains) | Q9UGI8 | No | 1 PET, 2 LIM |
| *TLN1* | talin 1 | Q9Y490 | Yes | 1 FERM, 1 Middle, 1 VBS, 1 C-term ILWEQ |
| *TRIP6* | thyroid hormone receptor interactor 6 | Q15654 | Yes | 3 LIM |
| *ZYX* | zyxin | Q15942 | Yes | 3 LIM |
| *ALYREF* | Aly/REF export factor | Q86V81 | Yes | 1 RRM, 1 FoP |
| *ANXA1* | annexin A1 | P04083 | No | 4 annexin |
| *BRIX1* | BRX1, biogenesis of ribosomes, homolog (S. cerevisiae) | Q8TDN6 | Yes | 1 Brix |
| *CALD1* | caldesmon 1 | Q05682 | Yes | 2 caldesmon |
| *CSK* | c-src tyrosine kinase | P41240 | No | 1 SH3, 1 SH2, 1 Kinase |
| *DDX27* | DEAD (Asp-Glu-Ala-Asp) box polypeptide 27 | Q96GQ7 | Yes | 1 DEAD, 1 helicase |
| *DIMT1* | DIM1 dimethyladenosine transferase 1 homolog (S. cerevisiae) | Q9UNQ2 | No | 1 RrnaAD |
| *DNAJB1* | DnaJ (Hsp40) homolog, subfamily B, member 1 | P25685 | Yes | 2 DnaJ |
| *FAU* | Finkel-Biskis-Reilly murine sarcoma virus (FBR-MuSV) ubiquitously expressed | P35544 | No | 1 Ubiquitin |
| *FBLIM1* | filamin binding LIM protein 1 | Q8WUP2 | Yes | 3 LIM |
| *FEN1* | flap structure-specific endonuclease 1 | P39748 | Yes | 2 XPG |
| *FLNC* | filamin C, gamma | Q14315 | Yes | 2 CH, 23 filamin repeats |
| *H1FX* | H1 histone family, member X | Q92522 | Yes | 1 linker histone |
| *HP1BP3* | heterochromatin protein 1, binding protein 3 | Q5SSJ5 | Yes | 3 linker histone |
| *IQGAP1* | IQ motif containing GTPase activating protein 1 | P46940 | Yes | 1 CH, 5 CC, 4 IQ, 2 GAP |
| *ITGB3* | integrin, beta 3 (platelet glycoprotein IIIa, antigen CD61) | P05106 | No |  |
| *LIMD1* | LIM domains containing 1 | Q9UGP4 | Yes | 3 LIM |
| *MRTO4* | mRNA turnover 4 homolog (S. cerevisiae) | Q9UKD2 | No | 1 RL10, 1 RL10 insert |
| *P4HB* | prolyl 4-hydroxylase, beta polypeptide | P07237 | No | 3 thiredoxin folds |
| *PDLIM1* | PDZ and LIM domain 1 | O00151 | Yes | 1 PDZ, 1 DUF, 1 LIM |
| *POLDIP3* | polymerase (DNA-directed), delta interacting protein 3 | Q9BY77 | Yes | 1 RRM |
| *PPIB* | peptidylprolyl isomerase B (cyclophilin B) | P23284 | No | 1 isomerase |
| *RPL23A* | ribosomal protein L23a | P62750 | No | 1 N-term, 1 L23 |
| *SIPA1* | signal-induced proliferation-associated 1 | Q96FS4 | Yes | 1 GAP, 1 PDZ, 1 CC |
| *SORBS1* | sorbin and SH3 domain containing 1 | Q9BX66 | Yes | 1 Sorb, 1 CC, 3 SH3 |
| *SORBS3* | sorbin and SH3 domain containing 3 | O60504 | Yes | 1 Sorb, 1 CC, 3 SH3 |
| *SYNCRIP* | synaptotagmin binding, cytoplasmic RNA interacting protein | O60506 | Yes | 1 hnRNP, 3 RRM |
| *TGFB1I1* | transforming growth factor beta 1 induced transcript 1 | O43294 | Yes | 1 Paxillin, 4 LIM |
| *TNS3* | tensin 3 | Q68CZ2 | Yes | 1 PTEN, 1 SH2, 1 PTB |
