# Supplementary material for: Synergistic phase separation of two pathways promotes integrin clustering and nascent adhesion formation
Source: eLife. 2022 Jan 20;11:e72588. doi: 10.7554/eLife.72588 (PMC8791637; doi:10.7554/eLife.72588)
Supplement: Supplementary file 6. — DNA used for expression in bacteria, SF9 cells (pFastBac vector), or mammalian cells (mEGFP-C1 vector). For bacteria and SF9 cell plasmids, the protein sequence of final protease-cleaved and purified protein is shown. For mammalian expression plasmids, the expressed protein sequence is shown. [file elife-72588-supp6.docx]

**Supplementary File 6.**

| **Protein** | **Sequence** | **Notes/Source** |
| --- | --- | --- |
| Lck | GAMDPEFANSLEPEPWFFKNLSRKDAERQLLAPGNTHGSFLIRESESTAGSFSLSVRDFDQNQGEVVKHYKIRNLDNGGFYISPRITFPGLHDLVRHYTNASDGLCTKLSRPCQTQKPQKPWWEDEWEVPRETLKLVERLGAGQFGEVWMGYYNGHTKVAVKSLKQGSMSPDAFLAEANLMKQLQHPRLVRLYAVVTQEPIYIITEYMENGSLVDFLKTPSGIKLNVNKLLDMAAQIAEGMAFIEEQNYIHRDLRAANILVSDTLSCKIADFGLARLIEDNEYTAREGAKFPIKWTAPEAINYGTFTIKSDVWSFGILLTEIVTHGRIPYPGMTNPEVIQNLERGYRMVRPDNCPEELYHLMMLCWKERPEDRPTFDYLRSVLDDFFTATEGQFQPQP | Human, 119–509, Y505F  Parent Vector: pFastBac HTa2  Tags: His_6_ (Tev cleavage)  Source: Case et al., 2019; PMID: 30846599 |
| p130Cas | GAMGSGTMKYLNVLAKALYDNVAESPDELSFRKGDIMTVLERDTQGLDGWWLCSLHGRQGIVPGNRLKILVGMYDKKPAAPGPGPPATPPQPQPSLPQGVHTPVPPASQYSPMLPTAYQPQPDNVYLVPTPSKTQQGLYQAPGPNPQFQSPPAKQTSTFSKQTPHHSFPSPATDLYQVPPGPGSPAQDIYQVPPSAGTGHDIYQVPPSLDTRSWEGTKPPAKVVVPTRVGQGYVYEASQAEQDEYDTPRHLLAPGSQDIYDVPPVRGLLPNQYGQEVYDTPPMAVKGPNGRDPLLDVYDVPPSVEKGLPPSNHHSVYDVPPSVSKDVPDGPLLREETYDVPPAFAKPKPFDPTRHPLILAAPPPDSPPAEDVYDVPPPAPDLYDVPPGLRRPGPGTLYDVPRERVLPPEVADGSVIDDGVYAVPPPAEREAPTDGKRLSASSTGSTRSSQSASSLEVVVPGREPLELEVAVETLARLQQGVSTTVAHLLDLVGSASGPGGWRSTSEPQEPPVQDLKAAVAAVHGAVHELLEFARSAVSSATHTSDRTLHAKLSRQLQKMEDVYQTLVVHGQVLDSGRGGPGFTLDDLDRLVACSRAVPEDAKQLASFLHGNASLLFRRTKAPGPGPEGSSSLHLNPTDKASSIQSRPLPSPPKFTSQDSPDGQYENSEGGWMEDYDYVHLQGKEEFEKTQKELLEKGNIVRQGKGQLELQQLKQFERLEQEVSRPIDHDLANWTPAQPLVPGRTGGLGPSDRQLLLFYLEQCEANLTTLTDAVDAFFTAVATNQPPKIFVAHSKFVILSAHKLVFIGDTLSRQAKAADVRSQVTHYSNLLCDLLRGIVATTKAAALQYPSPSAAQDMVDRVKELGHSTQQFRRVLGQLAAA | Rat, short isoform (NCBI XP_006255691.1)  Parent Vector: pFastBac Htb  Tags: His_6_ (Tev cleavage)  Source: Pellicena and Miller, 2001; PMID: 11389136 |
| N-WASP | GSEFKEKKKGKAKKKRAPPPPPPSRGGPPPPPPPPHSSGPPPPPARGRGAPPPPPSRAPTAAPPPPPPSRPGVVVPPPPPNRMYPHPPPALPSSAPSGPPPPPPLSMAGSTAPPPPPPPPPPPGPPPPPGLPSDGDHQVPASSGNKAALLDQIREGAQLKKVEQNSRPVSCSGRDALLDQIRQGIQLKSVSDGQESTPPTPAPTSGIVGALMEVMQKRSKAIHSSDEDEDDDDEEDFEDDDEWED | Rat, residues 183–193 fused to 273–501  Parent Vector: pMagic  Tag: His_6_ (Tev cleavage)  Source: Case et al., 2019; PMID: 30846599 |
| Nck | GHMCMAEEVVVVAKFDYVAQQEQELDIKKNERLWLLDDSKSWWRVRNSMNKTGFVPSNYVERKNSARKASIVKNLKDTLGIGKVKRKPSVPDSASPADDSFVDPGERLYDLNMPAYVKFNYMAEREDELSLIKGTKVIVMEKSSDGWWRGSYNGQVGWFPSNYVTEEGDSPLGDHVGSLSEKLAAVVNNLNTGQVLHVVQALYPFSSSNDEELNFEKGDVMDVIEKPENDPEWWKARKINMVGLVPKNYVTVMQNNPLTSGLEPSPPQSDYRPSLTGKFAGNPWYYGKVTRHQAEMALNERGHEGDFLIRDSESSPNDFSVSLKAQGKNKHFVQLKETVYSIGQRKFSTMEELVEHYKKAPIFTSEQGEKLYLVKHLS | Human, residues 1–377, with mutations: C139S, C232A, C266S, C340S  Parent Vector: ETpGEX  Tag: GST (Tev cleavage)  Source: Case et al., 2019; PMID: 30846599 |
| Nephrin | GHMHLYDEVERTFPPSGAWGPLYDEVQMGPWDLHWPEDTFQDPRGIYDQVAGDGSLEVLFQ | Human, residues 1174–1223, with mutations: Y1183F, Y1210F  Parent Vector: pMal-RTH  Tag: MBP (N-term, Tev cleavage); His_6_ (C-term, HRV 3C cleavage)  Source: Case et al., 2019; PMID: 30846599 |
| Kindlin | SMALDGIRMPDGCYADGTWELSVHVTDLNRDVTLRVTGEVHIGGVMLKLVEKLDVKKDWSDHALWWEKKRTWLLKTHWTLDKYGIQADAKLQFTPQHKLLRLQLPNMKYVKVKVNFSDRVFKAVSDICKTFNIRHPEELSLLKKPRDPTKKKKKKLDDQSEDEALELEGPLITPGSGSIYSSPGLYSKTMTPTYDAHDGSPLSPTSAWFGDSALSEGNPGILAVSQPITSPEILAKMFKPQALLDKAKINQGWLDSSRSLMEQDVKENEALLLRFKYYSFFDLNPKYDAIRINQLYEQAKWAILLEEIECTEEEMMMFAALQYHINKLSIMTSENHLNNSDKEVDEVDAALSDLEITLEGGKTSTILGDITSIPELADYIKVFKPKKLTLKGYKQYWCTFKDTSISCYKSKEESSGTPAHQMNLRGCEVTPDVNISGQKFNIKLLIPVAEGMNEIWLRCDNEKQYAHWMAACRLASKGKTMADSSYNLEVQNILSFLKMQHLNPDPQLIPEQITTDITPECLVSPRYLKKYKNKQITARILEAHQNVAQMSLIEAKMRFIQAWQSLPEFGITHFIARFQGGKKEELIGIAYNRLIRMDASTGDAIKTWRFSNMKQWNVNWEIKMVTVEFADEVRLSFICTEVDCKVVHEFIGGYIFLSTRAKDQNESLDEEMFYKLTSGWV | Parent Vector: pET28a  Tag: His_6_-Sumo  Source: This study. Modification of GST-Kindlin2 from Bledzka et al., 2010 (PMID: 20702409) |
| SNAP-Kindlin | SMDKDCEMKRTTLDSPLGKLELSGCEQGLHEIKLLGKGTSAADAVEVPAPAAVLGGPEPLMQATAWLNAYFHQPEAIEEFPVPALHHPVFQQESFTRQVLWKLLKVVKFGEVISYQQLAALAGNPAATAAVKTALSGNPVPILIPCHRVVSSSGAVGGYEGGLAVKEWLLAHEGHRLGKPGLGGSGSGSGGGGSSMALDGIRMPDGCYADGTWELSVHVTDLNRDVTLRVTGEVHIGGVMLKLVEKLDVKKDWSDHALWWEKKRTWLLKTHWTLDKYGIQADAKLQFTPQHKLLRLQLPNMKYVKVKVNFSDRVFKAVSDICKTFNIRHPEELSLLKKPRDPTKKKKKKLDDQSEDEALELEGPLITPGSGSIYSSPGLYSKTMTPTYDAHDGSPLSPTSAWFGDSALSEGNPGILAVSQPITSPEILAKMFKPQALLDKAKINQGWLDSSRSLMEQDVKENEALLLRFKYYSFFDLNPKYDAIRINQLYEQAKWAILLEEIECTEEEMMMFAALQYHINKLSIMTSENHLNNSDKEVDEVDAALSDLEITLEGGKTSTILGDITSIPELADYIKVFKPKKLTLKGYKQYWCTFKDTSISCYKSKEESSGTPAHQMNLRGCEVTPDVNISGQKFNIKLLIPVAEGMNEIWLRCDNEKQYAHWMAACRLASKGKTMADSSYNLEVQNILSFLKMQHLNPDPQLIPEQITTDITPECLVSPRYLKKYKNKQITARILEAHQNVAQMSLIEAKMRFIQAWQSLPEFGITHFIARFQGGKKEELIGIAYNRLIRMDASTGDAIKTWRFSNMKQWNVNWEIKMVTVEFADEVRLSFICTEVDCKVVHEFIGGYIFLSTRAKDQNESLDEEMFYKLTSGWV | Parent Vector: pET28a  Tag: His_6_-Sumo  Source: This study. Modification of GST-Kindlin2 from Bledzka et al., 2010 (PMID: 20702409) |
| $\beta1$ Integrin | GGGGGCKLLMIIHDRREFAKFEKEKMNAKWDTGENPIYKSAVTTVVNPKYEGK | Parent Vector: pMal  Tag: His_6_-MBP (N-term, Tev cleavage)  Source: This study; Synthesized DNA |
| $\beta1$ Integrin-GFP | GSGGGGCKLLMIIHDRREFAKFEKEKMNAKWDTGENPIYKSAVTTVVNPKYEGKGGSGGSGGSMVSKGEELFTGVVPILVELDGDVNGHKFSVSGEGEGDATYGKLTLKFICTTGKLPVPWPTLVTTLTYGVQCFSRYPDHMKQHDFFKSAMPEGYVQERTIFFKDDGNYKTRAEVKFEGDTLVNRIELKGIDFKEDGNILGHKLEYNYNSHNVYIMADKQKNGIKVNFKIRHNIEDGSVQLADHYQQNTPIGDGPVLLPDNHYLSTQSKLSKDPNEKRDHMVLLEFVTAAGITLGMDELYK | Parent Vector: pMal  Tag: His_6_-MBP (N-term, Tev cleavage)  Source: This study; Synthesized DNA |
| His_10_-$\beta1$ Integrin | GGSLEHHHHHHHHHHGGSCGGSGGSGGSGGSKLLMIIHDRREFAKFEKEKMNAKWDTGENPIYKSAVTTVVNPKYEGK | Parent Vector: pMal  Tag: MBP (N-term, Tev cleavage)  Source: This study; Synthesized DNA |
| Talin Head | MVALSLKISIGNVVKTMQFEPSTMVYDACRIIRERIPEAPAGPPSDFGLFLSDDDPKKGIWLEAGKALDYYMLRNGDTMEYRKKQRPLKIRMLDGTVKTIMVDDSKTVTDMLMTICARIGITNHDEYSLVRELMEEKKEEITGTLRKDKTLLRDEKKMEKLKQKLHTDDELNWLDHGRTLREQGVEEHETLLLRRKFFYSDQNVDSRDPVQLNLLYVQARDDILNGSHPVSFDKACEFAGFQCQIQFGPHNEQKHKAGFLDLKDFLPKEYVKQKGERKIFQAHKNCGQMSEIEAKVRYVKLARSLKTYGVSFFLVKEKMKGKNKLVPRLLGITKECVMRVDEKTKEVIQEWNLTNIKRWAASPKSFTLDFGDYQDGYYSVQTTEGEQIAQLIAGYIDIILKKKKSKDHFGLEGDEESTMLEDSVSPKKSTVLQAAAENLYF | Parent Vector: pET30b  Tag: His_6_ (C-term, Tev cleavage)  Source: This study |
| FAK | GAMGIRMAAAYLDPNLNHTPSSSTKTHLGTGMERSPGAMERVLKVFHHFESSSEPTTWASIIRHGDATDVRGIIQKIVDSHKVKHVACYGFRLSHLRSEEVHWLHVDMGVSSVREKYELAHPPEEWKYELRIRYLPKGFLNQFTEDKPTLNFFYQQVKSDYMQEIADQVDQEIALKLGCLEIRRSYWEMRGNALEKKSNYEVLEKDVGLKRFFPKSLLDSVKAKTLRKLIQQTFRQFANLNREESILKFFEILSPVYRFDKECFKCALGSSWIISVELAIGPEEGISYLTDKGCNPTHLADFNQVQTIQYSNSEDKDRKGMLQLKIAGAPEPLTVTAPSLTIAENMADLIDGYCRLVNGATQSFIIRPQKEGERALPSIPKLANSEKQGMRTHAVSVSETDDYAEIIDEEDTYTMPSTRDYEIQRERIELGRCIGEGQFGDVHQGVYLSPENPALAVAIKTCKNCTSDSVREKFLQEALTMRQFDHPHIVKLIGVITENPVWIIMELCTLGELRSFLQVRKYSLDLASLILYAYQLSTALAYLESKRFVHRDIAARNVLVSSNDCVKLGDFGLSRYMEDSTYYKASKGKLPIKWMAPESINFRRFTSASDVWMFGVCMWEILMHGVKPFQGVKNNDVIGRIENGERLPMPPNCPPTLYSLMTKCWAYDPSRRPRFTELKAQLSTILEEEKVQQEERMRMESRRQATVSWDSGGSDEAPPKPSRPGYPSPRSSEGFYPSPQHMVQTNHYQVSGYPGSHGIPAMAGSIYQGQASLLDQTELWNHRPQEMSMWQPSVEDSAALDLRGMGQVLPPHLMEERLIRQQQEMEEDQRWLEKEERFLKPDVRLSRGSIDREDGSFQGPTGNQHIYQPVGKPDPAAPPKKPPRPGAPGHLSNLSSISSPADSYNEGVKLQPQEISPPPTANLDRSNDKVYENVTGLVKAVIEMSSKIQPAPPEEYVPMVKEVGLALRTLLATVDETIPALPASTHREIEMAQKLLNSDLGELISKMKLAQQYVMTSLQQEYKKQMLTAAHALAVDAKNLLDVIDQARLKMLGQTRPH | Parent Vector: pFastBac  Tag: His_6_ (N-term, Tev cleavage)  Source: This study. FAK insert a gift from Liddington Lab, cloned into pFastBac plasmid. |
| Paxillin | GHMDDLDALLADLESTTSHISKRPVFLSEETPYSYPTGNHTYQEIAVPPPVPPPPSSEALNGTILDPLDQWQPSGSRFIHQQPQSSSPVYGSSAKTSSVSNPQDSVGSPCSRVGEEEHVYSFPNKQKSAEPSPTVMSTSLGSNLSELDRLLLELNAVQHNPPGFPADEANSSPPLPGALSPLYGVPETNSPLGGKAGPLTKEKPKRNGGRGLEDVRPSVESLLDELESSVPSPVPAITVNQGEMSSPQRVTSTQQQTRISASSATRELDELMASLSDFKFMAQGKTGSSSPPGGPPKPGSQLDSMLGSLQSDLNKLGVATVAKGVCGACKKPIAGQVVTAMGKTWHPEHFVCTHCQEEIGSRNFFERDGQPYCEKDYHNLFSPRCYYCNGPILDKVVTALDRTWHPEHFFCAQCGAFFGPEGFHEKDGKAYCRKDYFDMFAPKCGGCARAILENYISALNTLWHPECFVCRECFTPFVNGSFFEHDGQPYCEVHYHERRGSLCSGCQKPITGRCITAMAKKFHPEHFVCAFCLKQLNKGTFKEQNDKPYCQNCFLKLFC | Parent Vector: ETpGEX  Tag: GST (N-term, Tev cleavage)  Source: This study. Modification of Addgene Plasmid #50529. Paxillin cloned into ETpGEX vector. |
| SNAP-Paxillin | QGHMDKDCEMKRTTLDSPLGKLELSGCEQGLHEIKLLGKGTSAADAVEVPAPAAVLGGPEPLMQATAWLNAYFHQPEAIEEFPVPALHHPVFQQESFTRQVLWKLLKVVKFGEVISYQQLAALAGNPAATAAVKTALSGNPVPILIPCHRVVSSSGAVGGYEGGLAVKEWLLAHEGHRLGKPGLGGSGSGSGGGGSSHMDDLDALLADLESTTSHISKRPVFLSEETPYSYPTGNHTYQEIAVPPPVPPPPSSEALNGTILDPLDQWQPSGSRFIHQQPQSSSPVYGSSAKTSSVSNPQDSVGSPCSRVGEEEHVYSFPNKQKSAEPSPTVMSTSLGSNLSELDRLLLELNAVQHNPPGFPADEANSSPPLPGALSPLYGVPETNSPLGGKAGPLTKEKPKRNGGRGLEDVRPSVESLLDELESSVPSPVPAITVNQGEMSSPQRVTSTQQQTRISASSATRELDELMASLSDFKFMAQGKTGSSSPPGGPPKPGSQLDSMLGSLQSDLNKLGVATVAKGVCGACKKPIAGQVVTAMGKTWHPEHFVCTHCQEEIGSRNFFERDGQPYCEKDYHNLFSPRCYYCNGPILDKVVTALDRTWHPEHFFCAQCGAFFGPEGFHEKDGKAYCRKDYFDMFAPKCGGCARAILENYISALNTLWHPECFVCRECFTPFVNGSFFEHDGQPYCEVHYHERRGSLCSGCQKPITGRCITAMAKKFHPEHFVCAFCLKQLNKGTFKEQNDKPYCQNCFLKLFC | Parent Vector: ETpGEX  Tag: GST (N-term, Tev cleavage)  Source: This study. Modification of Addgene Plasmid #50529 |
| FAK W266A | GAMGIRMAAAYLDPNLNHTPSSSTKTHLGTGMERSPGAMERVLKVFHHFESSSEPTTWASIIRHGDATDVRGIIQKIVDSHKVKHVACYGFRLSHLRSEEVHWLHVDMGVSSVREKYELAHPPEEWKYELRIRYLPKGFLNQFTEDKPTLNFFYQQVKSDYMQEIADQVDQEIALKLGCLEIRRSYWEMRGNALEKKSNYEVLEKDVGLKRFFPKSLLDSVKAKTLRKLIQQTFRQFANLNREESILKFFEILSPVYRFDKECFKCALGSSAIISVELAIGPEEGISYLTDKGCNPTHLADFNQVQTIQYSNSEDKDRKGMLQLKIAGAPEPLTVTAPSLTIAENMADLIDGYCRLVNGATQSFIIRPQKEGERALPSIPKLANSEKQGMRTHAVSVSETDDYAEIIDEEDTYTMPSTRDYEIQRERIELGRCIGEGQFGDVHQGVYLSPENPALAVAIKTCKNCTSDSVREKFLQEALTMRQFDHPHIVKLIGVITENPVWIIMELCTLGELRSFLQVRKYSLDLASLILYAYQLSTALAYLESKRFVHRDIAARNVLVSSNDCVKLGDFGLSRYMEDSTYYKASKGKLPIKWMAPESINFRRFTSASDVWMFGVCMWEILMHGVKPFQGVKNNDVIGRIENGERLPMPPNCPPTLYSLMTKCWAYDPSRRPRFTELKAQLSTILEEEKVQQEERMRMESRRQATVSWDSGGSDEAPPKPSRPGYPSPRSSEGFYPSPQHMVQTNHYQVSGYPGSHGIPAMAGSIYQGQASLLDQTELWNHRPQEMSMWQPSVEDSAALDLRGMGQVLPPHLMEERLIRQQQEMEEDQRWLEKEERFLKPDVRLSRGSIDREDGSFQGPTGNQHIYQPVGKPDPAAPPKKPPRPGAPGHLSNLSSISSPADSYNEGVKLQPQEISPPPTANLDRSNDKVYENVTGLVKAVIEMSSKIQPAPPEEYVPMVKEVGLALRTLLATVDETIPALPASTHREIEMAQKLLNSDLGELISKMKLAQQYVMTSLQQEYKKQMLTAAHALAVDAKNLLDVIDQARLKMLGQTRPH | Parent Vector: pFastBac  Tag: His_6_ (N-term, Tev cleavage)  Source: This study. |
| FAK Fus | GAMGIRMAAAYLDPNLNHTPSSSTKTHLGTGMERSPGAMERVLKVFHHFESSSEPTTWASIIRHGDATDVRGIIQKIVDSHKVKHVACYGFRLSHLRSEEVHWLHVDMGVSSVREKYELAHPPEEWKYELRIRYLPKGFLNQFTEDKPTLNFFYQQVKSDYMQEIADQVDQEIALKLGCLEIRRSYWEMRGNALEKKSNYEVLEKDVGLKRFFPKSLLDSVKAKTLRKLIQQTFRQFANLNREESILKFFEILSPVYRFDKECFKCALGSSWIISVELAIGPEEGISYLTDKGCNPTHLADFNQVQTIQYSNSEDKDRKGMLQLKIAGAPEPLTVTAPSLTIAENMADLIDGYCRLVNGATQSFIIRPQKEGERALPSIPKLANSEKQGMRTHAVSVSETDDYAEIIDEEDTYTMPSTRDYEIQRERIELGRCIGEGQFGDVHQGVYLSPENPALAVAIKTCKNCTSDSVREKFLQEALTMRQFDHPHIVKLIGVITENPVWIIMELCTLGELRSFLQVRKYSLDLASLILYAYQLSTALAYLESKRFVHRDIAARNVLVSSNDCVKLGDFGLSRYMEDSTYYKASKGKLPIKWMAPESINFRRFTSASDVWMFGVCMWEILMHGVKPFQGVKNNDVIGRIENGERLPMPPNCPPTLYSLMTKCWAYDPSRRPRFTELKAQLSTILEEEKVQQEE**ASNDYTQQATQSYGAYPTQPGQGYSQQSSQPYGQQSYSGYSQSTDTSGYGQSSYSSYGQSQNTGYGTQSTPQGYGSTGGYGSSQSSQSSYGQQSSYPGYGQQPAPSSTSGSYGSSSQSSSYGQPQSGSYSQQPSYGGQQQSYGQQQSYNPPQGYGQQNQYNSSSGGGGGGGGGGNYGQDQSSMSSGGGSGGGYGNQDQSGGGGSGGYGQQDRG**PTANLDRSNDKVYENVTGLVKAVIEMSSKIQPAPPEEYVPMVKEVGLALRTLLATVDETIPALPASTHREIEMAQKLLNSDLGELISKMKLAQQYVMTSLQQEYKKQMLTAAHALAVDAKNLLDVIDQARLKMLGQTRPH | Parent Vector: pFastBac  Tag: His_6_ (N-term, Tev cleavage)  Source: This study.  Linker in **BOLD.** |
| FAK Pyk2 | GAMGIRMAAAYLDPNLNHTPSSSTKTHLGTGMERSPGAMERVLKVFHHFESSSEPTTWASIIRHGDATDVRGIIQKIVDSHKVKHVACYGFRLSHLRSEEVHWLHVDMGVSSVREKYELAHPPEEWKYELRIRYLPKGFLNQFTEDKPTLNFFYQQVKSDYMQEIADQVDQEIALKLGCLEIRRSYWEMRGNALEKKSNYEVLEKDVGLKRFFPKSLLDSVKAKTLRKLIQQTFRQFANLNREESILKFFEILSPVYRFDKECFKCALGSSWIISVELAIGPEEGISYLTDKGCNPTHLADFNQVQTIQYSNSEDKDRKGMLQLKIAGAPEPLTVTAPSLTIAENMADLIDGYCRLVNGATQSFIIRPQKEGERALPSIPKLANSEKQGMRTHAVSVSETDDYAEIIDEEDTYTMPSTRDYEIQRERIELGRCIGEGQFGDVHQGVYLSPENPALAVAIKTCKNCTSDSVREKFLQEALTMRQFDHPHIVKLIGVITENPVWIIMELCTLGELRSFLQVRKYSLDLASLILYAYQLSTALAYLESKRFVHRDIAARNVLVSSNDCVKLGDFGLSRYMEDSTYYKASKGKLPIKWMAPESINFRRFTSASDVWMFGVCMWEILMHGVKPFQGVKNNDVIGRIENGERLPMPPNCPPTLYSLMTKCWAYDPSRRPRFTELKAQLSTILEEEKVQQEE**QERNARYRTPKILEPTAFQEPPPKPSRPKYRPPPQTNLLAPKLQFQVPEGLCASSPTLTSPMEYPSPVNSLHTPPLHRHNVFKRHSMREEDFIQPSSREEAQQLWEAEKVKMRQILDKQQKQMVEDYQWLRQEEKSLDPMVYMNDKSPLTPEKEVGYLEFTGPPQKPPRLGAQSI**PTANLDRSNDKVYENVTGLVKAVIEMSSKIQPAPPEEYVPMVKEVGLALRTLLATVDETIPLLPASTHREIEMAQKLLNSDLGELINKMKLAQQYVMTSLQQEYKKQMLTAAHALAVDAKNLLDVIDQARLKMLGQTRPH | Parent Vector: pFastBac  Tag: His_6_ (N-term, Tev cleavage)  Source: This study.  Linker in **BOLD.** |
| FAK C-term | **S**ERMRMESRRQATVSWDSGGSDEAPPKPSRPGYPSPRSSEGFYPSPQHMVQTNHYQVSGYPGSHGIPAMAGSIYQGQASLLDQTELWNHRPQEMSMWQPSVEDSAALDLRGMGQVLPPHLMEERLIRQQQEMEEDQRWLEKEERFLKPDVRLSRGSIDREDGSFQGPTGNQHIYQPVGKPDPAAPPKKPPRPGAPGHLSNLSSISSPADSYNEGVKLQPQEISPPPTANLDRSNDKVYENVTGLVKAVIEMSSKIQPAPPEEYVPMVKEVGLALRTLLATVDETIPALPASTHREIEMAQKLLNSDLGELISKMKLAQQYVMTSLQQEYKKQMLTAAHALAVDAKNLLDVIDQARLKMLGQTRPH | Parent Vector: pET28a  Tag: His_6_-Sumo  Source: This study. |
| GFP-FAK WT | MVSKGEELFTGVVPILVELDGDVNGHKFSVSGEGEGDATYGKLTLKFICTTGKLPVPWPTLVTTLTYGVQCFSRYPDHMKQHDFFKSAMPEGYVQERTIFFKDDGNYKTRAEVKFEGDTLVNRIELKGIDFKEDGNILGHKLEYNYNSHNVYIMADKQKNGIKVNFKIRHNIEDGSVQLADHYQQNTPIGDGPVLLPDNHYLSTQSKLSKDPNEKRDHMVLLEFVTAAGITLGMDELYKSGLRSGGSGSGMAAAYLDPNLNHTPSSSTKTHLGTGMERSPGAMERVLKVFHHFESSSEPTTWASIIRHGDATDVRGIIQKIVDSHKVKHVACYGFRLSHLRSEEVHWLHVDMGVSSVREKYELAHPPEEWKYELRIRYLPKGFLNQFTEDKPTLNFFYQQVKSDYMQEIADQVDQEIALKLGCLEIRRSYWEMRGNALEKKSNYEVLEKDVGLKRFFPKSLLDSVKAKTLRKLIQQTFRQFANLNREESILKFFEILSPVYRFDKECFKCALGSSWIISVELAIGPEEGISYLTDKGCNPTHLADFNQVQTIQYSNSEDKDRKGMLQLKIAGAPEPLTVTAPSLTIAENMADLIDGYCRLVNGATQSFIIRPQKEGERALPSIPKLANSEKQGMRTHAVSVSETDDYAEIIDEEDTYTMPSTRDYEIQRERIELGRCIGEGQFGDVHQGVYLSPENPALAVAIKTCKNCTSDSVREKFLQEALTMRQFDHPHIVKLIGVITENPVWIIMELCTLGELRSFLQVRKYSLDLASLILYAYQLSTALAYLESKRFVHRDIAARNVLVSSNDCVKLGDFGLSRYMEDSTYYKASKGKLPIKWMAPESINFRRFTSASDVWMFGVCMWEILMHGVKPFQGVKNNDVIGRIENGERLPMPPNCPPTLYSLMTKCWAYDPSRRPRFTELKAQLSTILEEEKVQQEERMRMESRRQATVSWDSGGSDEAPPKPSRPGYPSPRSSEGFYPSPQHMVQTNHYQVSGYPGSHGIPAMAGSIYQGQASLLDQTELWNHRPQEMSMWQPSVEDSAALDLRGMGQVLPPHLMEERLIRQQQEMEEDQRWLEKEERFLKPDVRLSRGSIDREDGSFQGPTGNQHIYQPVGKPDPAAPPKKPPRPGAPGHLSNLSSISSPADSYNEGVKLQPQEISPPPTANLDRSNDKVYENVTGLVKAVIEMSSKIQPAPPEEYVPMVKEVGLALRTLLATVDETIPALPASTHREIEMAQKLLNSDLGELISKMKLAQQYVMTSLQQEYKKQMLTAAHALAVDAKNLLDVIDQARLKMLGQTRPH | Parent Vector: mEGFP-C1 (Addgene Plasmid #54759, Mammalian Expression)  Source: This study. |
| GFP-FAK W266A | MVSKGEELFTGVVPILVELDGDVNGHKFSVSGEGEGDATYGKLTLKFICTTGKLPVPWPTLVTTLTYGVQCFSRYPDHMKQHDFFKSAMPEGYVQERTIFFKDDGNYKTRAEVKFEGDTLVNRIELKGIDFKEDGNILGHKLEYNYNSHNVYIMADKQKNGIKVNFKIRHNIEDGSVQLADHYQQNTPIGDGPVLLPDNHYLSTQSKLSKDPNEKRDHMVLLEFVTAAGITLGMDELYKSGLRSGGSGSGMAAAYLDPNLNHTPSSSTKTHLGTGMERSPGAMERVLKVFHHFESSSEPTTWASIIRHGDATDVRGIIQKIVDSHKVKHVACYGFRLSHLRSEEVHWLHVDMGVSSVREKYELAHPPEEWKYELRIRYLPKGFLNQFTEDKPTLNFFYQQVKSDYMQEIADQVDQEIALKLGCLEIRRSYWEMRGNALEKKSNYEVLEKDVGLKRFFPKSLLDSVKAKTLRKLIQQTFRQFANLNREESILKFFEILSPVYRFDKECFKCALGSSAIISVELAIGPEEGISYLTDKGCNPTHLADFNQVQTIQYSNSEDKDRKGMLQLKIAGAPEPLTVTAPSLTIAENMADLIDGYCRLVNGATQSFIIRPQKEGERALPSIPKLANSEKQGMRTHAVSVSETDDYAEIIDEEDTYTMPSTRDYEIQRERIELGRCIGEGQFGDVHQGVYLSPENPALAVAIKTCKNCTSDSVREKFLQEALTMRQFDHPHIVKLIGVITENPVWIIMELCTLGELRSFLQVRKYSLDLASLILYAYQLSTALAYLESKRFVHRDIAARNVLVSSNDCVKLGDFGLSRYMEDSTYYKASKGKLPIKWMAPESINFRRFTSASDVWMFGVCMWEILMHGVKPFQGVKNNDVIGRIENGERLPMPPNCPPTLYSLMTKCWAYDPSRRPRFTELKAQLSTILEEEKVQQEERMRMESRRQATVSWDSGGSDEAPPKPSRPGYPSPRSSEGFYPSPQHMVQTNHYQVSGYPGSHGIPAMAGSIYQGQASLLDQTELWNHRPQEMSMWQPSVEDSAALDLRGMGQVLPPHLMEERLIRQQQEMEEDQRWLEKEERFLKPDVRLSRGSIDREDGSFQGPTGNQHIYQPVGKPDPAAPPKKPPRPGAPGHLSNLSSISSPADSYNEGVKLQPQEISPPPTANLDRSNDKVYENVTGLVKAVIEMSSKIQPAPPEEYVPMVKEVGLALRTLLATVDETIPALPASTHREIEMAQKLLNSDLGELISKMKLAQQYVMTSLQQEYKKQMLTAAHALAVDAKNLLDVIDQARLKMLGQTRPH | Parent Vector: mEGFP-C1 (Addgene Plasmid #54759, Mammalian Expression)  Source: This study. |
| Paxillin LD | GHMDDLAALLADLESTTSHISKRPVFLSEETPYSYPTGNHTYQEIAVPPPVPPPPSSEALNGTILDPLDQWQPSGSRFIHQQPQSSSPVYGSSAKTSSVSNPQDSVGSPCSRVGEEEHVYSFPNKQKSAEPSPTVMSTSLGSNLSELARLLLELNAVQHNPPGFPADEANSSPPLPGALSPLYGVPETNSPLGGKAGPLTKEKPKRNGGRGLEDVRPSVESLLAELESSVPSPVPAITVNQGEMSSPQRVTSTQQQTRISASSATRELAELMASLSDFKFMAQGKTGSSSPPGGPPKPGSQLASMLGSLQSDLNKLGVATVAKGVCGACKKPIAGQVVTAMGKTWHPEHFVCTHCQEEIGSRNFFERDGQPYCEKDYHNLFSPRCYYCNGPILDKVVTALDRTWHPEHFFCAQCGAFFGPEGFHEKDGKAYCRKDYFDMFAPKCGGCARAILENYISALNTLWHPECFVCRECFTPFVNGSFFEHDGQPYCEVHYHERRGSLCSGCQKPITGRCITAMAKKFHPEHFVCAFCLKQLNKGTFKEQNDKPYCQNCFLKLFC | Parent Vector: ETpGEX  Tag: GST (N-term, Tev cleavage)  Source: This Study; Synthesized DNA |
| Paxillin DP | GHMDDLDALLADLESTTSHISKRPVFLSEETPYSYPTGNHTYQEIAVPPPVPPPPSSEALNGTILDPLDQWQPSGSRFIHQQPQSSSPVYGSSAKTSSVSNPQDSVGSPCSRVGEEEHVYSFPNKQKSAEPSPTVMSTSLGSNLSELDRLLLELNAVQHNPPGFPADEANSSPPLPGALSPLYGVPETNSPLGGKAGPLTKEKPKRNGGRGLEDVRPSVESLLDELESSVPSPVPAITVNQGEMSSPQRVTSTQQQTRISASSATRELDELMASLSDFKFMAQGKTGSSSPPGGPPKPGSQLDSMLGSLQSDLNKLGVATVAKMDDLDALLADLESTTSHISKRPVFLSEETPYSYPTGNHTYQEIAVPPPVPPPPSSEALNGTILDPLDQWQPSGSRFIHQQPQSSSPVYGSSAKTSSVSNPQDSVGSPCSRVGEEEHVYSFPNKQKSAEPSPTVMSTSLGSNLSELDRLLLELNAVQHNPPGFPADEANSSPPLPGALSPLYGVPETNSPLGGKAGPLTKEKPKRNGGRGLEDVRPSVESLLDELESSVPSPVPAITVNQGEMSSPQRVTSTQQQTRISASSATRELDELMASLSDFKFMAQGKTGSSSPPGGPPKPGSQLDSMLGSLQSDLNKLGVATVAKGVCGACKKPIAGQVVTAMGKTWHPEHFVCTHCQEEIGSRNFFERDGQPYCEKDYHNLFSPRCYYCNGPILDKVVTALDRTWHPEHFFCAQCGAFFGPEGFHEKDGKAYCRKDYFDMFAPKCGGCARAILENYISALNTLWHPECFVCRECFTPFVNGSFFEHDGQPYCEVHYHERRGSLCSGCQKPITGRCITAMAKKFHPEHFVCAFCLKQLNKGTFKEQNDKPYCQNCFLKLFC | Parent Vector: ETpGEX  Tag: GST (N-term, Tev cleavage)  Source: This Study; Synthesized DNA |
| GFP-Paxillin WT | MVSKGEELFTGVVPILVELDGDVNGHKFSVSGEGEGDATYGKLTLKFICTTGKLPVPWPTLVTTLTYGVQCFSRYPDHMKQHDFFKSAMPEGYVQERTIFFKDDGNYKTRAEVKFEGDTLVNRIELKGIDFKEDGNILGHKLEYNYNSHNVYIMADKQKNGIKVNFKIRHNIEDGSVQLADHYQQNTPIGDGPVLLPDNHYLSTQSKLSKDPNEKRDHMVLLEFVTAAGITLGMDELYKSGLRSGSGGSMDDLDALLADLESTTSHISKRPVFLSEETPYSYPTGNHTYQEIAVPPPVPPPPSSEALNGTILDPLDQWQPSGSRFIHQQPQSSSPVYGSSAKTSSVSNPQDSVGSPCSRVGEEEHVYSFPNKQKSAEPSPTVMSTSLGSNLSELDRLLLELNAVQHNPPGFPADEANSSPPLPGALSPLYGVPETNSPLGGKAGPLTKEKPKRNGGRGLEDVRPSVESLLDELESSVPSPVPAITVNQGEMSSPQRVTSTQQQTRISASSATRELDELMASLSDFKFMAQGKTGSSSPPGGPPKPGSQLDSMLGSLQSDLNKLGVATVAKGVCGACKKPIAGQVVTAMGKTWHPEHFVCTHCQEEIGSRNFFERDGQPYCEKDYHNLFSPRCYYCNGPILDKVVTALDRTWHPEHFFCAQCGAFFGPEGFHEKDGKAYCRKDYFDMFAPKCGGCARAILENYISALNTLWHPECFVCRECFTPFVNGSFFEHDGQPYCEVHYHERRGSLCSGCQKPITGRCITAMAKKFHPEHFVCAFCLKQLNKGTFKEQNDKPYCQNCFLKLFC | Parent Vector: mEGFP-C1 (Addgene Plasmid #54759, Mammalian Expression)  Source: This study. Modification of Addgene Plasmid #50529 |
| GFP-Paxillin DP | MVSKGEELFTGVVPILVELDGDVNGHKFSVSGEGEGDATYGKLTLKFICTTGKLPVPWPTLVTTLTYGVQCFSRYPDHMKQHDFFKSAMPEGYVQERTIFFKDDGNYKTRAEVKFEGDTLVNRIELKGIDFKEDGNILGHKLEYNYNSHNVYIMADKQKNGIKVNFKIRHNIEDGSVQLADHYQQNTPIGDGPVLLPDNHYLSTQSKLSKDPNEKRDHMVLLEFVTAAGITLGMDELYKSGLRSGSGGSMDDLDALLADLESTTSHISKRPVFLSEETPYSYPTGNHTYQEIAVPPPVPPPPSSEALNGTILDPLDQWQPSGSRFIHQQPQSSSPVYGSSAKTSSVSNPQDSVGSPCSRVGEEEHVYSFPNKQKSAEPSPTVMSTSLGSNLSELDRLLLELNAVQHNPPGFPADEANSSPPLPGALSPLYGVPETNSPLGGKAGPLTKEKPKRNGGRGLEDVRPSVESLLDELESSVPSPVPAITVNQGEMSSPQRVTSTQQQTRISASSATRELDELMASLSDFKFMAQGKTGSSSPPGGPPKPGSQLDSMLGSLQSDLNKLGVATVAKMDDLDALLADLESTTSHISKRPVFLSEETPYSYPTGNHTYQEIAVPPPVPPPPSSEALNGTILDPLDQWQPSGSRFIHQQPQSSSPVYGSSAKTSSVSNPQDSVGSPCSRVGEEEHVYSFPNKQKSAEPSPTVMSTSLGSNLSELDRLLLELNAVQHNPPGFPADEANSSPPLPGALSPLYGVPETNSPLGGKAGPLTKEKPKRNGGRGLEDVRPSVESLLDELESSVPSPVPAITVNQGEMSSPQRVTSTQQQTRISASSATRELDELMASLSDFKFMAQGKTGSSSPPGGPPKPGSQLDSMLGSLQSDLNKLGVATVAKGVCGACKKPIAGQVVTAMGKTWHPEHFVCTHCQEEIGSRNFFERDGQPYCEKDYHNLFSPRCYYCNGPILDKVVTALDRTWHPEHFFCAQCGAFFGPEGFHEKDGKAYCRKDYFDMFAPKCGGCARAILENYISALNTLWHPECFVCRECFTPFVNGSFFEHDGQPYCEVHYHERRGSLCSGCQKPITGRCITAMAKKFHPEHFVCAFCLKQLNKGTFKEQNDKPYCQNCFLKLFC | Parent Vector: mEGFP-C1 (Addgene Plasmid #54759, Mammalian Expression)  Source: This Study; Synthesized DNA |
| CasY15F | GAMGSGTMKYLNVLAKALYDNVAESPDELSFRKGDIMTVLERDTQGLDGWWLCSLHGRQGIVPGNRLKILVGMYDKKPAAPGPGPPATPPQPQPSLPQGVHTPVPPASQYSPMLPTAYQPQPDNVFLVPTPSKTQQGLFQAPGPNPQFQSPPAKQTSTFSKQTPHHSFPSPATDLFQVPPGPGSPAQDIFQVPPSAGTGHDIFQVPPSLDTRSWEGTKPPAKVVVPTRVGQGYVYEASQAEQDEFDTPRHLLAPGSQDIFDVPPVRGLLPNQYGQEVFDTPPMAVKGPNGRDPLLDVFDVPPSVEKGLPPSNHHSVFDVPPSVSKDVPDGPLLREETFDVPPAFAKPKPFDPTRHPLILAAPPPDSPPAEDVFDVPPPAPDLFDVPPGLRRPGPGTLFDVPRERVLPPEVADGSVIDDGVFAVPPPAEREAPTDGKRLSASSTGSTRSSQSASSLEVVVPGREPLELEVAVETLARLQQGVSTTVAHLLDLVGSASGPGGWRSTSEPQEPPVQDLKAAVAAVHGAVHELLEFARSAVSSATHTSDRTLHAKLSRQLQKMEDVYQTLVVHGQVLDSGRGGPGFTLDDLDRLVACSRAVPEDAKQLASFLHGNASLLFRRTKAPGPGPEGSSSLHLNPTDKASSIQSRPLPSPPKFTSQDSPDGQYENSEGGWMEDYDYVHLQGKEEFEKTQKELLEKGNIVRQGKGQLELQQLKQFERLEQEVSRPIDHDLANWTPAQPLVPGRTGGLGPSDRQLLLFYLEQCEANLTTLTDAVDAFFTAVATNQPPKIFVAHSKFVILSAHKLVFIGDTLSRQAKAADVRSQVTHYSNLLCDLLRGIVATTKAAALQYPSPSAAQDMVDRVKELGHSTQQFRRVLGQLAAA | Parent Vector: pFastBac  Tag: His_6_ (N-term, Tev cleavage)  Source: This study. |
